# Tick-borne encephalitis virus (TBEV) prevalence in field-collected ticks (*Ixodes ricinus*) and phylogenetic, structural and virulence analysis in a TBE high-risk endemic area in southwestern Germany

**DOI:** 10.1186/s13071-020-04146-7

**Published:** 2020-06-11

**Authors:** Daniela Ott, Kristina Ulrich, Philip Ginsbach, Rainer Öhme, Oswinde Bock-Hensley, Ulrich Falk, Martina Teinert, Thorsten Lenhard

**Affiliations:** 1 Neuroinfectious Diseases Group, Department of Neurology, University Hospital Heidelberg, University of Heidelberg, Im Neuenheimer Feld 350, 69120 Heidelberg, Germany; 2 Institute of Aquaculture, University of Stirling, Stirling, FK9 4LA Scotland, UK; 3 Mathematics, University of Oxford, Oxford, OX1 2JD UK; 4 Molecular Biology Laboratory, Landesgesundheitsamt Stuttgart, Nordbahnhofstraße 135, 70191 Stuttgart, Germany; 5 Gesundheitsamt Rhein-Neckarkreis, Kurfürsten-Anlage 38-40, 69115 Heidelberg, Germany; 6 Gesundheitsamt Odenwaldkreis, Michelstädter Str. 12, 64711 Erbach, Germany; 7 Gesundheitsamt Neckar-Odenwaldkreis, Neckarelzer Str. 7, 74821 Mosbach, Germany

**Keywords:** Tick-borne encephalitis, Germany, Risk-area, *Ixodes ricinus*, Flavivirus, TBEV prevalence, Phylogeny, Envelope protein

## Abstract

**Background:**

Tick-borne encephalitis (TBE) is the most common viral CNS infection with incidences much higher than all other virus infections together in many risk areas of central and eastern Europe. The Odenwald Hill region (OWH) in southwestern Germany is classified as a TBE risk region and frequent case numbers but also more severe infections have been reported within the past decade. The objective of the present study was to survey the prevalence of tick-borne encephalitis virus (TBEV) in *Ixodes ricinus* and to associate TBEV genetic findings with TBE infections in the OWH.

**Methods:**

Ticks were collected by the flagging methods supported by a crowdsourcing project implementing the interested public as collectors to cover completely and collect randomly a 3532 km^2^ area of the OWH TBE risk region. Prevalence of TBEV in *I. ricinus* was analysed by reversed transcription quantitative real-time PCR. Phylogeographic analysis was performed to classify OWH TBEV isolates within a European network of known TBEV strains. Mutational sequence analysis including 3D modelling of envelope protein pE was performed and based on a clinical database, a spatial association of TBE case frequency and severity was undertaken.

**Results:**

Using the crowd sourcing approach we could analyse a total of 17,893 ticks. The prevalence of TBEV in *I. ricinus* in the OWH varied, depending on analysed districts from 0.12% to 0% (mean 0.04%). Calculated minimum infection rate (MIR) was one decimal power higher. All TBEV isolates belonged to the European subtype. Sequence analysis revealed a discontinuous segregation pattern of OWH isolates with two putative different lineages and a spatial association of two isolates with increased TBE case numbers as well as exceptional severe to fatal infection courses.

**Conclusions:**

TBEV prevalence within the OWH risk regions is comparatively low which is probably due to our methodological approach and may more likely reflect prevalence of natural TBEV foci. As for other European regions, TBEV genetics show a discontinuous phylogeny indicating among others an association with bird migration. Mutations within the pE gene are associated with more frequent, severe and fatal TBE infections in the OWH risk region.
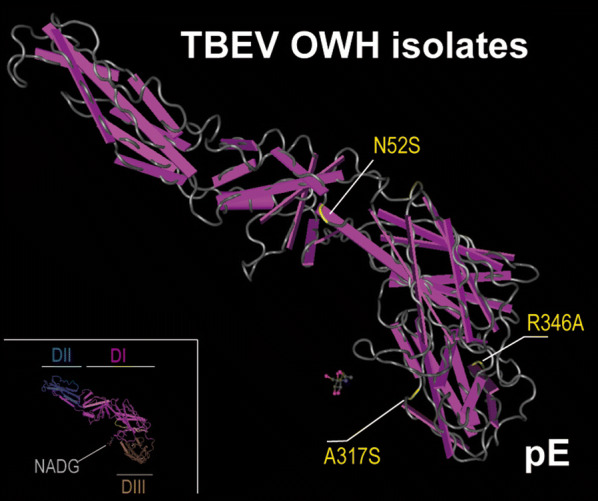

## Background

Tick-borne encephalitis (TBE) is the numerical most relevant tick-borne CNS-infection in central and eastern Europe. In some endemic regions of Europe, the subnational incidence is much higher (up to 29/100,000 population per year) than meningoencephalitis caused by all other sporadic viruses (14/100,000 population per year) [1–5]. The causative pathogen is tick-borne encephalitis virus (TBEV), belonging to the tick-borne encephalitis virus serocomplex (among others further members: louping ill-, Powassan-, Omsk hemorrhagic fever virus), a member of the *Flavivirus* family [6]. Three TBEV subtypes mainly cause TBE along the 8 °C isotherm in Eurasia and Japan with specific geographical but also overlapping distribution patterns have been described: Far-Eastern subtype (TBEV-Fe), Siberian subtype (TBEV-Sib) and European subtype (TBEV-Eu) [7]. Two additional subtypes have been currently identified in the Himalayan and Baikal regions [8, 9].

TBEV-Eu is less virulent compared to TBEV-Sib and especially to the TBEV-Fe. However, mortality of brain infections range between 1.0–3.6% and approximately 40% of infected patients suffer from more or less long-term sequelae [10–14].

In Europe, more than 12,500 TBE cases are reported annually from 23 risk countries [1]. The overall European incidence fluctuates at 0.4/100,000 population per year, but the geographical annual notification rates differ significantly and range up to 15 cases per 100,000 population in Lithuania on the national and up to 29 cases per 100,000 population on the subnational level e.g. in Slovenia. In Germany, despite annual fluctuations, an overall continuous increase in TBE infections was recognized in the past two decades. The numerical most important risk areas reporting TBE infections are Bavaria and Baden-Württemberg located in the south and south-west [15].

TBEV is a zoonotic pathogen and is maintained in the ecosystem by cycling within competent reservoir hosts, especially small rodents, e.g. the yellow-necked mouse (*Apodemus flavicollis*) but also in wild ungulate animals, wild boar or foxes [16]. As a critical link within the cycle, different tick species act as transmitting vectors. In central Europe, the main vector is the castor bean tick *Ixodes ricinus*, and in Finland and the Baltics states *Ixodes persulcatus* too [17]. The prevalence of TBEV within its vector has been analysed in the past in Germany in several foci mainly in Bavaria [18–20]. In general, the incidence in *I. ricinus* populations is low and varies between 0.1 and 5.0% [21]. At a spatial scale within risk areas, it has been assumed that the prevalence of TBEV-infected ticks and reservoir hosts show a highly patchy pattern and these patches have been referred to as foci [22–24]. Up to today it is not well understood how natural foci behave in time and space. Detailed analysis on circulating viruses and associated sequence data has been carried out in the past ten years especially in Bavarian foci, and revealed evidence for a discontinuous geographical and evolutionary distribution pattern of TBEV isolates. Biogenic (e.g. deer roar and bird migration) but also anthropogenic (e.g. small rodents on long-haul lorry transport) spread patterns of viruses are thought to support these findings [20, 25].

The TBEV virion contains a single, positive strand RNA genome (11 kb) coding for one large polyglycoprotein that is post-translationally processed into three structural proteins: capsid protein (pC), envelope protein (pE) and a matrix protein (pM). The remaining RNA codes for seven non-structural proteins (NS1, 2a, 2b, 3, 4a, 4b and 5) [26, 27]. It is suggested that pE plays a major role for the infection of mammalian and tick cells and is identified as a critical factor in defining virulence. Long-term analysis over 44 years from well-defined foci has suggested that the pE gene is subjected to purifying selection [28]. However, humans are dead end hosts and do not contribute to this evolutionary process. Thus, less is known on natural occurring mutagenesis affecting virulence in humans. Point mutations leading to amino acid exchanges in the prM, pE and NS1 glycoprotein were identified in Zika virus, yellow fever virus and West Nile virus, also members of the flavivirus family, that interfere with neuro-invasiveness and neurotoxicity [29].

The present study aims to investigate systematically the prevalence of TBEV in *I. ricinus* tick populations in the Odenwald hill region (OWH), a well characterized TBE risk region of south-western Germany concerning epidemiology and neurological infection courses [10]. More detailed phylogenetic analysis of TBEV isolates should clarify their relationship to the European TBE virus network. Furthermore, by performing sequence analysis and 3D structural modeling of pE of TBEV isolates, we intended to associate relevant mutations with the geographical distribution pattern and the clinical course of TBE cases in the Odenwald hill risk region.

## Methods

### Tick collection in the field

**Fig. 1:**
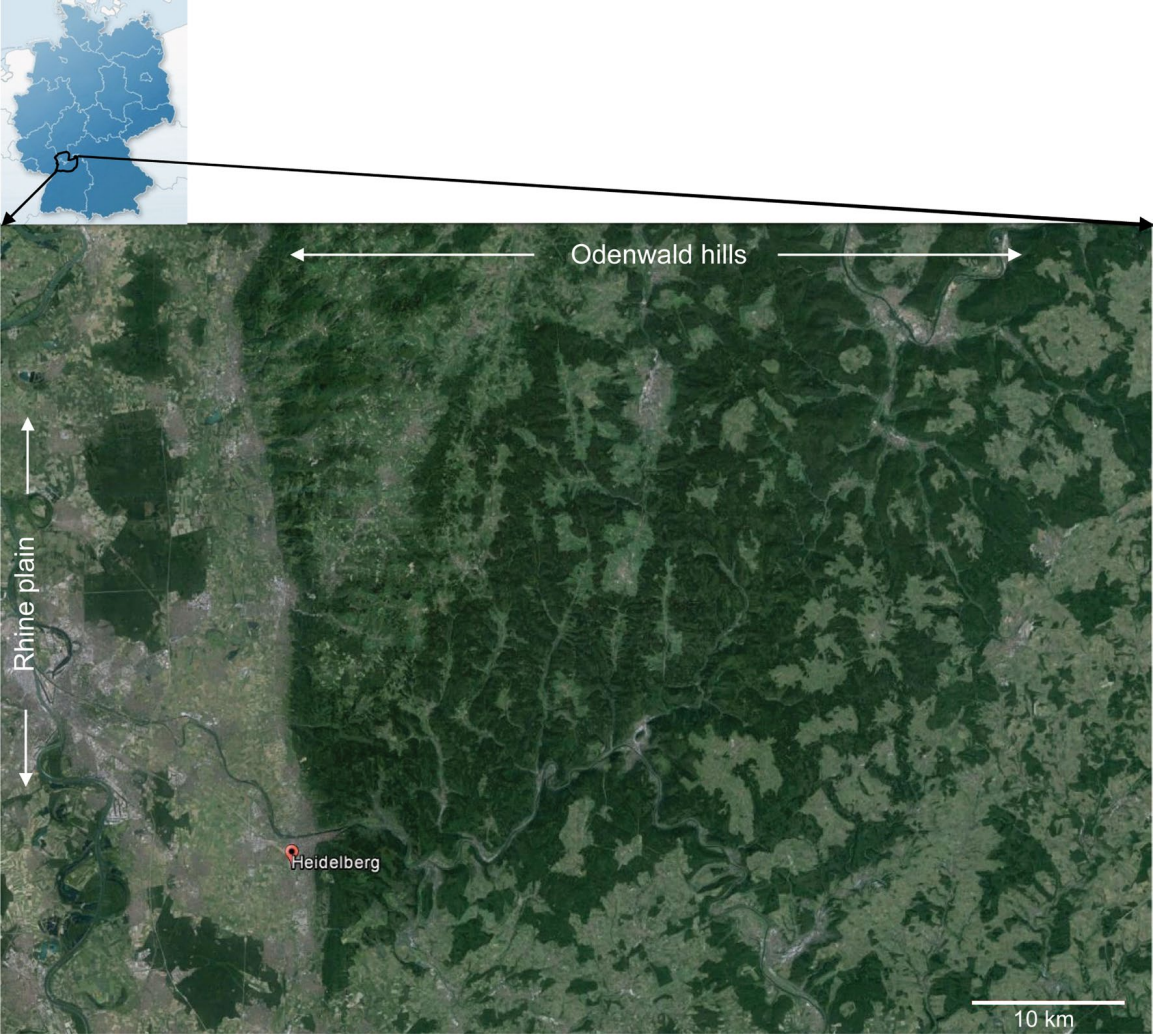
The OWH: a high risk TBE endemic area. The image at top left depicts a schematic map of Germany with the borders of the federal states traced in light blue. The OWH risk area is located in south-western Germany and belongs to the federal states of Baden-Württemberg and Hessen (bold-framed in black). The main image shows a satellite view of the OWH, the wooded area to the east of the Rhine plain. The city of Heidelberg is located at the western fringe of the risk area where the hills meet the Rhine plain (Source: Google Maps, Google 2014©, Kartendaten© 2014 GeoBasis-DE/BKB; GNU Free Documentation License, version 1.2, Copyright© 2002 Free Software Foundation)

Ticks were collected between September 2011 and May 2013 in the risk area of the Odenwald hills (OWH) (Fig. 1). To collect ticks randomly, rather than related to reported TBE cases, we covered the OWH with a raster divided into 5 × 5 km^2^. From every raster at least from one randomly selected area (100 × 100 m) ticks were collected using the flagging method [30, 31]. The number of ticks within every single area was defined as at least 100 nymphs of *I. ricinus* plus by-catch of adult male and female *Ixodes* and other tick species. Ticks were pooled according to developmental stage and sex (10 nymphs per tube and 5 adult female or male ticks per tube) and were stored at − 80 °C until further analyses. Others species (*Dermacentor reticulatus*) were analyzed individually.

Since this region covers a surface area of 3,531.52 km^2^, we recruited interested members of the public for tick collection. Therefore, we undertook a public call in the media (radio, print and posting in public authority buildings) and performed an information event at every district public health office dealing with tick-borne disease biology, epidemiology and prevention. Thus, we recruited potential collectors that were trained in small groups in the field in using the flags and in acquiring basic knowledge on how to identify ticks, tick stages and tick habitats. Every participant had to prove that they had received a complete TBE vaccination. The trained collectors selected at least one or more raster squares, collected ticks autonomously in a randomly selected natural area (see above) within a given raster and sent the collected ticks by mail, already pooled in 2 ml safe-lock tubes. The ticks were always sent with a fresh green leaf within every tube to ensure atmospheric humidity. Numbers of ticks within a pool as well as the quality and viability of ticks were checked before freezing. Furthermore, exact coordinates from every area were collected either using GPS devices or Google Earth to trace subsequent TBEV positive foci.

### RNA extraction and one-step real-time RT-PCR

Two microliters safe lock tubes containing the frozen ticks/tick pools were mixed with ice-cold 400 µl homogenization buffer (MEM Earle, Biochrom Berlin, Germany; gentamycin 100 µg/ml, Biochrom; RNAsin 40 U/µl, Fermentas, Karlsruhe, Germany). Three stainless steel beads (dm = 3 mm) per tube were added and the tick shells were cracked using a tissue lyser (TissueLYser II, Qiagen, Hilden, Germany) at 30 Hz for 10 min at RT. Pool/tick homogenates were centrifuged at 1000× *g* for 10 min at RT. Then, 200 µl of the homogenate was subjected to RNA extraction, whilst the remaining homogenates were stored at − 80 °C for eventual virus propagation. RNA extraction was performed using silica-based columns adapted for virus nucleic acids (Viral RNA Mini Kit, Qiagen) according to the manufacturer guidelines. RNA was eluted in 50 µl/sample ddH_2_O/0.04% sodium azide.

To identify TBEV positive pools/ticks, a real-time one-step RT-PCR was performed, using the TaqMan®RNA-to-CT™ 1-Step-Kit (Applied Biosystems, Life Technologies, Darmstadt, Germany) with the following primers: (forward: 5′-GGG CGG TTC TTG TTC TCC-3′; reverse: 5′-ACA CAT CAC CTC CTT GTC AGA CT-3′). The primers cover a 68-bp fragment of the 3′ non-coding region of the TBEV genome as was described for the first time by Schwaiger & Cassinotti [32]. RNA extracts from BHC TBEV culture supernatants (strain: Hypr, AC U39292) at serial dilutions of 4 ng up to 0.7 pg RNA/RXN at the highest dilution were always amplified in parallel as positive controls to avoid false negative results [33]. Distilled water was used as a negative control. A sequence-specific TagMan® oligonucleotide-probe labeled with 6-Carboxy-Fluorescin at the 5′ terminus as a fluorescent reporter, and 6-Carboxyl-tetrametyl-rhodamin at the 3′OH terminus as a quencher (5′-6FAM-TGA GCC ACC ATC ACC CAG ACA CA-TAMRA-3′) was used to produce specific, quantitative amplification signals [32]. Five microliters of RNA per sample were subjected to each reaction. Cycling conditions were used as followed: RT reaction at 48 °C for 20 min; POL activation at 95 °C for 10 min; followed by 50 cycles with melting at 95 °C for 15 s followed by annealing and extension at 60 °C for 60 s. Cycling was performed on an ABI-7300s real-time Cycler (Life technologies, Darmstadt, Germany).

To validate the quality of RNA extraction from the ticks, every 10th probe of TBEV negative ticks/pools were subjected to a qualitative RT-PCR detecting a 200 bp fragment of *I. ricinus 18S*-rDNA, using the following primers: (forward: 5′-AGA TCG TTT CTT CCT ACT TGG A-3′; reverse: 5′-ACC TAC CAT CGA CAG TTG ATA-3′) [34]. The products were amplified (2 µl cDNA sample) using a two-step method with AmpliTaq®360 DNA Polymerase (Life Technologies, Karlsruhe, Germany) after reverse transcription of 100 ng RNA with M-MLV reverse polymerase (Promega, Mannheim, Germany) according to the manufacturer guidelines. Reaction conditions were as follows: RT: 37 °C for 60 min; PCR: activation 93 °C for 3 min, melting 95 °C for 30 s, annealing 49 °C for 30 s, extension 72 °C for 30 s, 40 cycles. Amplicons were separated by agarose gel electrophoresis and visualized by SYBR® Safe DNA gel stain (Life Technologies).

To validate the identified positive pools/ticks, a confirmation PCR was performed in a second, independent laboratory (Molecular Biology Laboratory, State Public Health Office, Stuttgart, Baden-Württemberg). Therefore, our identified positive pools and randomly selected negative pools were sent to the validation laboratory and were reanalyzed anonymously.

### Statistical analysis

As a commonly used method to analyse the prevalence of pathogens within vectors, we have screened collected *I. ricinus* ticks as pools. However, using this approach, it is no longer possible to trace whether a positive pool contains only a single infected tick or more. The minimum infection rate (MIR) is based on the assumption that every positive pool contains only a single infected tick. MIR was calculated as follows: MIR = Number of positive pools/Total no. of specimens tested × 1000 [35].

### TBEV pE gene PCR sequencing

TBEV pE gene product was sequenced directly from PCR products *via* endpoint detection. Therefore, 5 µl of RNA extracts from TBEV-positive pools or positive single ticks verified by the above described TBEV real-time RT-PCR were subjected to a one-step RT-PCR using the combination of the highly thermostable superscript III reverse transcriptase and the highly specific and sensitive Platinum Taq Polymerase combined with the high fidelity proofreading activity of *Pyrococcus* GB-D polymerase (SuperScript® III One-Step RT-PCR System with Platinum® *Taq* High Fidelity DNA Polymerase; Life Technologies) with an error frequency of 1.8 ± 0.3 × 10^−6^ (manufacturer data). In the first instance, three oligos that prime at position 885 (5′-GGT TAC CGT TGT GTG GTT GAC C-3′) and at position 2571 (5′-CTC CGG GTA GTA GGC ATA ATT G-3′, 5′-CTC CGG GTA GTA TGC ATA ATT G-3′) within the TBEV genome were used as PCR and sequencing primers, resulting in product spanning 88 bp upstream and 111 bp downstream, respectively of the *de facto* pE gene. As reverse primers an oligo pair was used that recognizes all known TBEV strains including the Hypr [19, 36]. The expected 1686 bp product was separated by electrophoresis on a 1.5% agarose gel (UltraPure™ Agarose; Invitrogen, Darmsatdt, Karlsruhe), cut under UV-light and purified using a gel-extraction kit (QIAquick Gel Extraction Kit, Qiagen). PCR products were eluted from the columns with 50 µl AVE buffer (containing 10 mM TRIS-HCL, pH = 7.8). For further sequencing, 15 µl at a concentration of 10 ng/µl + 2 µl primer (10 µM) were sent to a sequencing service (Eurofins Genomics, Ebersberg, Germany; ISO accrediting number 17025). The samples were analyzed by capillary (non-radioactive) Sanger sequencing on an ABI3730xl DNA analyzer in a 96-well format. In order to verify the quality and ensure the correctness of the sequencing results, internal DNA markers were put on each sequencing plate on predefined plate positions. Upon completion of the sequencing run, the marker sequences were evaluated with regard to the correct plate positions, reference sequences, signal strength and chromatogram quality obtained. For sequence reactions the above described PCR primers were used in all cases. The retrieved sequences (FastA format) were cut to 1488 bp, the precise length of the pE gene, and further processed for alignment, phylogenetic and 3D structural analysis.

### Phylogenetic analysis of TBEV

Sequences from collected isolates and 22 reference sequences from the NCBI GenBank database were used for phylogenetic analysis. The Louping ill virus (GenBank: KF056331), a member of the TBE complex but distant to TBEV, was chosen as an outgroup reference. Within the TBEV, isolates from three Far Eastern subtypes, two Siberian subtypes and 16 European subtypes were chosen. The latter were selected in order that reference isolates are represented from geographically close and distant areas in Europe (South and West Germany, North Switzerland, South Switzerland, Austria, Czech Republic, Slovenia, Sweden and Finland).

TBEV pE DNA sequences were aligned in the first instance using the multiple sequence alignment algorithm CLUSTALW (SDSC Biology WorkBench, San Diego Supercomputer Center, http://workbench.sdsc.edu/) [37] and in a second instance the sequences using the default settings of the Clustal Omega (version 1.0.3) and then performed the phylogenetic analysis with GARLI (Genetic Algorithm for Rapid Likelihood Inference) version 2.01, generating 100 bootstrap replicates [38]. The SumTrees program of the DendroPy Phylogenetic Computing Library (version 3.12.0) was utilized to summarize the bootstrap trees into one consensus tree, which was visualized using the FigTree software [39].

### Bayesian evolutionary analysis by sampling trees

The BEAST package v1.8.3 was used to analyze the complete pE sequence (1482 bp) of the 7 OWH isolates and 51 reference TBEV strains (see GenBank accession numbers within the figures) [40]. The date flagged strains allowed to infer a maximum clade credibility (MCC) tree with dated tips and internal nodes using a MCMC Bayesian approach with the following parameters: 10,000,000 chains per cycle with every 1000th chain being recorded, using the GTR substitution model under consideration of mutation rate of 8.0 × 10^−4^ substitutions per site, as previously calculated for TBEV by Weidmann et al. [20], an uncorrelated relaxed clock and a constant size tree without rooting [20, 25]. The tree was viewed and edited using FigTree v1.4.0 [40].

### MJ network analysis of TBEV

Reduced median joining (MJ) can describe linear inheritance patterns of viruses. Thus, we performed MJ network analysis to analyze the grade of continuity of the newly sampled OWH isolates. A network was constructed that include our OWH isolates and the already introduced (see above) 51 reference strains of southern, northern and eastern TBEV strains. Analysis based on pE gene sequences of a 1488-character alignment stripped of all homogeneous characters using SPLITS TREE 4.0 [20] with Epsilon 1 and 2000 Spring embedded iterations.

### 3D structural analysis of TBEV pE

We used the known X-ray crystal structure of the N-terminal ectodomains of TBEV pE (residues 1–395; protein DataBank: 1SVB) to create a homolog model of TBEV pE [41]. pE cDNA sequences were translated into amino acid sequence using the DNA-to-Protein translation tool (http://bio.lundberg.gu.se/edu/translat.html). The frame resulting in a complete amino acid sequence without preterm chain termination (in all cases frame 1) was chosen for further analyses. On amino acid level, a sequence comparison and alignment of the OWH isolates with two reference strains (strain Neudörfl, GenBank: U27495; strain Hypr, GenBank: U39292) was performed with the protein CLUST W algorithm (SDSC Biology WorkBench, San Diego Supercomputer Center, http://workbench.sdsc.edu/) [37]. Next, protein homology and structure prediction analyses were performed with the HHpred tool, a part of the open source package HHsuite (Bioinformatics ToolKit, Max-Planck Institute for Developmental Biology, Tübingen, Germany; http://toolkit.tuebingen.mpg.de/sections/search) [42, 43]. Three-dimensional structural modeling and visualizing was performed with the Molecular Graphic and 3D Viewer tool (NCBI Structure Summary MMDB; http://www.ncbi.nlm.nih.gov/Structure/MMDB/mmdb.shtml) [44].

## Results

### Systematic tick field collection using the public as multipliers

Ticks were collected by flagging in autumn 2011, spring to autumn 2012 and spring 2013. To address systematic TBEV prevalence analysis in the Odenwald hills (OWH) risk regions, ticks were collected randomly rather than related to reported TBE cases. Therefore, we covered the OWH with a raster divided into grid squares of 5 × 5 km^2^. Within every grid square at least from one randomly selected area of 100 × 100 m ticks were collected. Since the monitored OWH risk regions cover a surface area of 3531.52 km^2^, we started a call in the media to recruit for and educate the interested public in the flagging method as multipliers. GPS devices were used to collect geographical coordinates from flacked areas to be able to track later subsequent TBEV isolates. In total 61 public collectors were trained and took part in the project. With the support of these collectors, ticks within a total number of 136 grid squares were collected and a final grid square coverage of 74.2 to 78.4% was reached in three of four districts. In one district (KB) with lower coverage, local hunters instead of the interested public were trained and performed the collection. The remaining grid squares were covered by members of the projects, mainly by DO. In total 17,893 ticks were collected and further processed for TBEV analysis. Regarding the tick species, 99.46% of all collected ticks were from the genus *Ixodes* and all were identified as *Ixodes ricinus*, although no further efforts were made to separate into *Ixodes inopinatus* that has been recently described also outside the Mediterranean region and may also be present in the OWH [45]. Eighty-four ticks (0.54%) were identified as *Dermacentor reticulatus*. All *D. reticulatus* individuals were collected in the district Kreis Bergstaße (KB), in forests belonging to the ecotype of alluvial forest near to the River Rhine. In addition, 340 engorged ticks (all *I. ricinus*) mainly preying on deer were collected during drive hunting from 3 of 4 districts.

### TBEV prevalence in the OWH high-risk region

Collected ticks from the OWH risk region were tested for TBEV mRNA using real-time PCR with TBEV-specific primers and internal fluorescent-labeled oligonucleotide probes according to the original protocol of Schwaiger & Cassinotti [32]. Nymphs of *I. ricinus* were pooled in groups of 10 ticks and adults were separated by sex into pools of 5 ticks. Engorged *I. ricinus* ticks, copulating ticks and adult *D. reticulatus* were analysed individually. Thus, 2228 tick pools were analysed in total, see Additional file 1: Table S1 for details. In 6 *I. ricinus* pools and 1 *D. reticulatus*, the 68-bp specific TBEV-pE fragment was detected. For further validation, an aliquot of the 6 positive pools, RNA from the *D. reticulatus* extract and 20 randomly selected negative pools were sent blinded to a reference laboratory (Molecular Biology Laboratory, Federal State Health Department, Stuttgart, Germany) to confirm our results. The pool-associated TBEV-positive *I. ricinus* ticks and the TBEV-positive *D. reticulatus* ticks were detected in three of the four federal districts. The deduced overall TBEV minimum infection rate (MIR) within the four districts of the OWH regions was 0.4% (Table 1). The highest MIR (1.2%) was recorded in the district Bergstraße (KB) and the lowest detectable MIR (0.3%.) was recorded in the district Odenwaldkreis (OK). In the district Neckar-Odenwaldkreis none of the 692 pools were positive. The prevalence within TBEV-positive grid square areas of 100 × 100 m varied from 0.6% up to 4.7%. The MIR was 4.7-fold lower in nymphs (0.3%) compared to adult females (1.4%) and half as high compared to male ticks (0.6%). For the sake of completeness, the MIR was highest in *D. reticulatus* (11.9%), but this should be interpreted with caution because of a possible bias due to the low sample size. The Cq-values for the OWH isolates ranged between 16.5–33.7 (mean 25.1). As positive controls, strain Hypr (RNA from cell culture supernatants) was amplificated in parallel as serial dilutions covering Cq-values from 21.9 (lowest dilution) up to 37.4 (highest dilution). TBEV mRNA could not be detected in any of the engorged and copulating ticks. Table 1 summarizes the prevalence data in detail.

**Table 1:** Prevalence and minimum infections rate of TBEV in *Ixodes ricinus* in the Odenwald Hill risk region

District	Tick stage	No. of ticks	Positive pools	MIR	95% CI
Odenwaldkreis (OK)	I.r. N	3052	1	0.3	0.298–0.302
	I.r. A_f_	285	0	0	na
	I.r. A_m_	370	0	0	na
	I.r. A_c_	18	0	0	na
	D.r. A	0	0	0	na
	Total	3725	1	0.3	0.299–0.301
Neckar-Odenwald-Kreis (NOK)	I.r. N	4650	0	0	na
	I.r. A_f_	401	0	0	na
	I.r. A_m_	467	0	0	na
	I.r. A_c_	6	0	0	na
	D.r. A	0	0	0	na
	Total	5524	0	0	na
Rhein-Neckarkreis (RNK)	I.r. N	5965	3	0.5	0.498–0.502
	I.r. A_f_	459	1	2.2	2.162–2.237
	I.r. A_m_	518	0	0	na
	I.r. A_c_	20	0	0	na
	D.r. A	0	0	0	na
	Total	6962	4	0.6	0.599–0.601
Kreis Bergstraße (KB)	I.r. N	1054	0	0	na
	I.r. A_f_	254	0	0	na
	I.r. A_m_	290	1	3.5	3.394–3.606
	I.r. A_c_	0	0	0	na
	D.r. A	84	1	11.9	11.622–12.178
	Total	1682	2	1.2	1.19–1.21
Total OWH	I.r. N	14721	4	0.3	0.2996–0.3004
	I.r. A_f_	1399	1	0.7	0.698–0.702
	I.r. A_m_	1645	1	0.6	0.598–0.602
	I. r.A_c_	44	0	0	na
	D.r. A	84	1	11.9	11.622–12.178
	Total	17,893	7	0.4	0.3998–0.4002

*Abbreviations*: MIR, minimum infection rate depicted in %; 95% CI, 95% confidence interval; I.r., *Ixodes ricinus*; N, nymphs; A_f_, adult females; A_m_, adult males; A_c_, coupling male and female; D.r., adult *Dermacentor reticularis*; OWH, Odenwald hill region; na, not available

### Sequence analysis of the glycoprotein E gene

All pools that tested positive for TBEV were successfully sequenced for the whole pE gene. The sequences have been submitted to the NCBI GenBank database (accession numbers are provided in Additional file 2: Alignment S1). Compared to the reference strains Neudörfl and Hypr [33], the sequenced 1488-bp pE fragment of our seven isolates contains in total 61 nucleotide exchanges accounting for a sequence variability of 4.2% in total. On the single isolate level, the sequence variability was as follows: TBEV_KB_D1A2M7, 1.69%; OKA4N5, 1.35%; RNK_F7A2N1, 1.35%; RNK_F7A2N1, 1.42%; KB_C3d3, 1.21%; RNK_C4A5N8, 1.01%; and RNK_C4A5N9, 1.15%. The majority comprised synonymous nucleotide exchanges but seven were non-synonymous exchanges (11.5% of all exchanges; 0.47% of total pE). Additional file 2: Alignment S1 shows a Clustal W sequence alignment of pE of the OWH isolates compared to the reference strains Neudoerfl and Hypr.

### Phylogenetic analysis

Phylogenetic analysis revealed that five of seven OWH isolates (isolates of *I. ricinus*, district RNK and one isolate of *D. reticulatus*, district KB) were closely related whereas two isolates of *I. ricinus* (district OK and KB) were more distant as depicted with a Levensthein plot (see Additional file 3: Figure S1 for detailed illustration). All OWH isolates belong to the European subtype of TBEV and were clearly more distant from Far Eastern and Siberian type TBEV as well as from louping ill TBE strains that were used as the outgroup as depicted with a Levenshtein plot (blue, European-type TBEV; red, out-roots; Additional file 4: Figure S2)

### Bayesian phylodynamic analysis

**Fig. 2:**
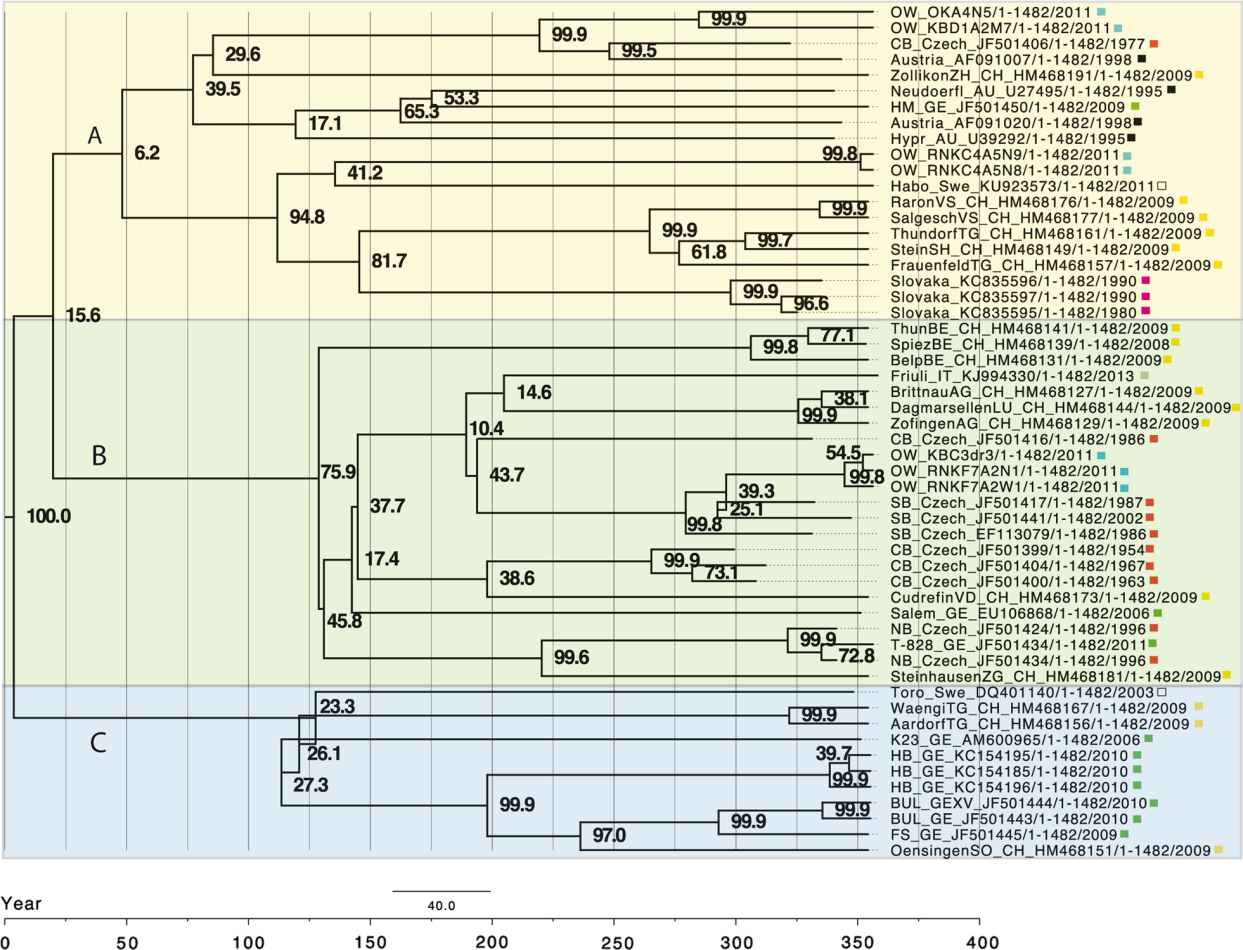
Phylogenetic classification of OWH TBEV isolates within a European-type network. The unrooted tree was calculated using BEAST with 10,000,000 chains per cycle with every 1000th chain being recorded. The mutation rate of 8.0 × 10^−4^ substitutions per site was considered according to Weidmann et al. [20, 25]. Posterior probability values are shown at corresponding nodes in percent. The scale-bar represents 40.0 substitutions per nucleotide site. Samples are marked by countries for visual clarity (blue, OWH samples; green, other German isolates; red, Czech Republic; yellow, Switzerland; black, Austria; pink, Slovakia; white, Sweden; grey, Italy). Nomenclature of an individual European isolate includes GenBank accession number, number of base pairs analyzed (1482) and year of isolation at the end. Internal nomenclature of OWH isolates (blue) in chronological order: OW, Odenwald hills; followed by district shortcut; geographical coordinates in terms of grid square; A, number of collected area; N, nymph, W, female, M, male, for *I. ricinus*; dr, *D. reticulatus*; 1482, number of base pairs analyzed; year of isolation

To analyze and visualize TBEV evolution of a European TBEV network and to integrate our OWH isolates within this network, a different approach was applied using the BEAST package v1.8.3 software. Applying the GTR substitution model under consideration of mutation rate of 8.0 × 10^−4^ substitutions per site, it was shown that the OWH isolates (blue label) segregated into two main groups that split approximately 350 years ago (Fig. 2). Group A comprised of strains (blue label) from eastern parts of the OHW (districts KB, OW and RNK) and strains from Switzerland (yellow label), from Austria (black label), Slovakia (purple label) and one each from central Bohemia (Czech Republic), Sweden and Germany, respectively (Fig. 2a). However, Group B comprised of OWH strains from the district RNK and KB (blue label), that are located westward close to or within the Rhine plain. These OWH strains segregated with strains from the Czech Republic (red label), strains from Switzerland (yellow label), two South German strains (green label), one strain from the Rhine plain (T-828, green label) and one from Italy (grey label) (Fig. 2b). Interestingly, the isolates coming from OWH, were not grouping closely with other German strains (green label). None of our strains segregated into a third group (Group C) that represents strains from Bavaria, Switzerland and one from Sweden (Fig. 2c).

### Median joining (MJ) network analysis

**Fig. 3:**
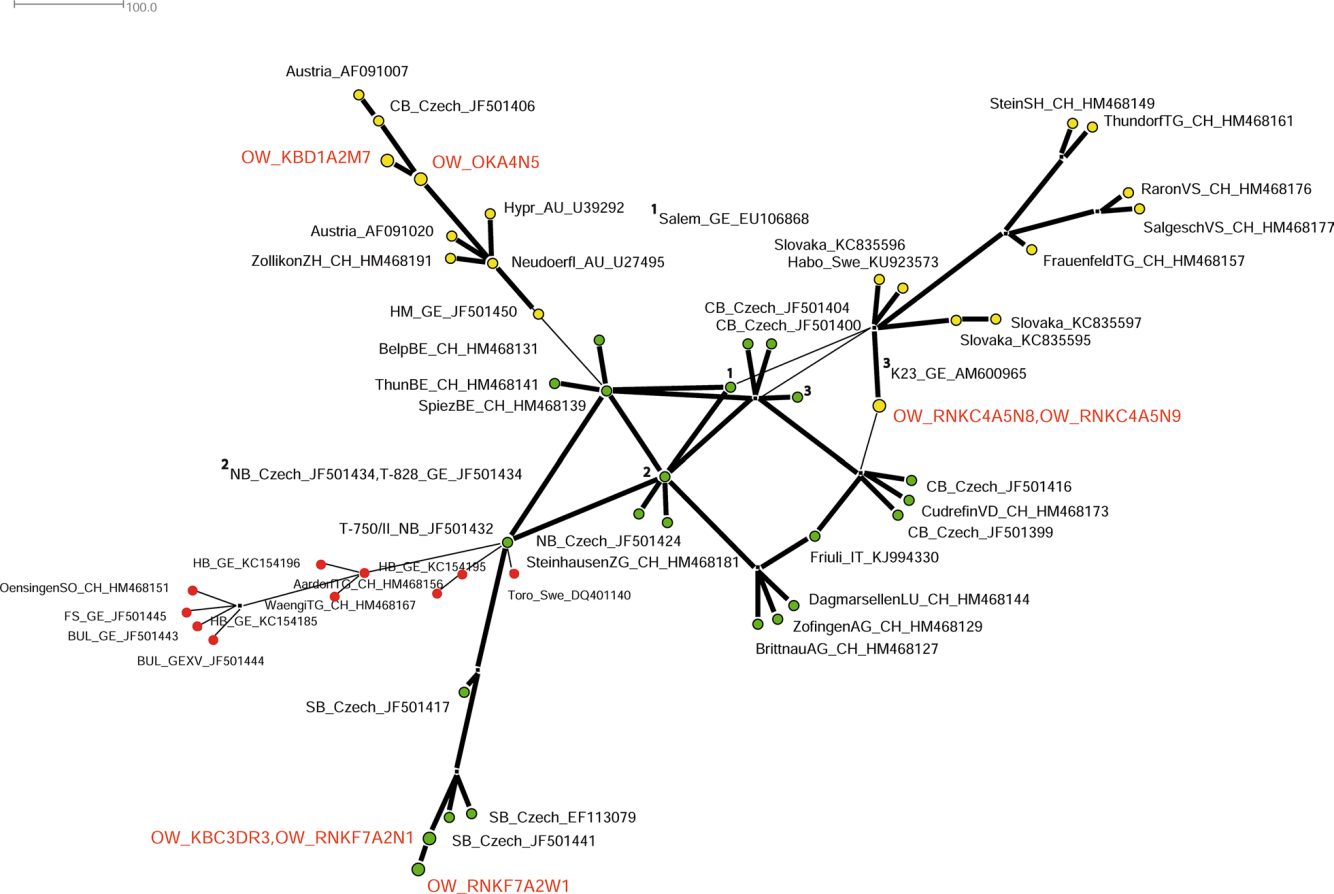
Reduced MJ network plot of a 1488-character dataset of 54 TBEV pE sequences including our OWH isolates in red letters. Two different lineages (yellow and green circles) could be differentiated according to the MCC tree in Fig. 2. A third lineage that is not represented in the OWH is indicated in red circles

We were interested whether our isolates from the OWH have evolved from a common offspring or may show a discontinuous inheritance pattern. To address this question, we performed MJ network analysis, since this approach can reveal linear inheritance patterns of viral sequences. The resulting MJ network based on the MCC tree (see Fig. 2) shows a discontinuous pattern of lineages A and B with five of seven OWH isolates to be more closely related to Austrian, Swiss and Slovakian strains, but also to one Swedish and one German isolate from the Rhine plain (yellow circle), whereas two more distant OWH isolates from districts OK and KB seem to be mainly related to Czech strains but also to a considerable number of Swiss strains (green circle) (Fig. 3).

### 3D structure analysis of pE

**Fig. 4:**
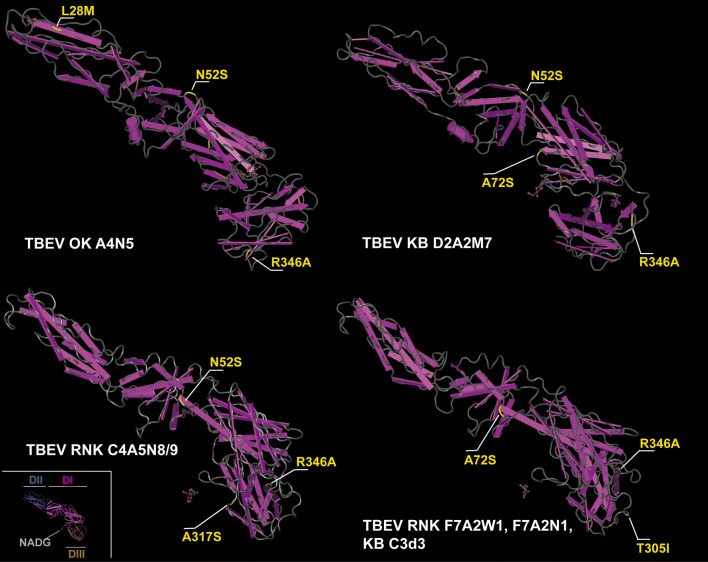
3D reconstruction of TBEV pE and position of mutations. Inset (left, bottom): the tertiary structure of the TBEV envelope protein and its organization into the ectodomains I-III (pink, blue, brown), stem and transmembrane region (yellow) is depicted. Main figure: four different pE clusters representing common and variant types of mutations. The amino acid residues and side chain substitutions are indicated in yellow; the respective OWH TBEV isolates linked with pE clusters are indicated in white

We investigated 3D structural analysis of pE to visualize the non-synonymous mutations of the OWH isolates and to extrapolate putative functional effects of these mutations within the protein structure and putative interference with regard to critical binding sides. pE consists of a large 395 amino acid (AS) N-terminal ectodomaine and a C-terminal trans-membrane anchor, both linked by an alpha-helical stem [26, 27]. The ectodomain consists of three domains: the central domain (DI; purple), the fusion domain (DII; blue), and the lateral domain (DIII; brown) (Fig. 4, insert). The stem consists of three helices (H1, H2 and H3). The two trans-membrane helices (T1 and T2) are inserted in the viral membrane. Based on the known crystal structure of TBEV pE we created models of the OWH isolates by applying the HHsuite software package and the Molecular Graphic and 3D Viewer (Bioinformatics ToolKit, MPI Tübingen). In the OWH isolates all amino acid substitutions mapped to the ectodomains but not to the C-terminal part or the stem (Fig. 4). One amino acid substitute was located in DII, two in DI and three in DIII. The mutations and the associated biochemical properties are summarized in Additional file 5: Table S2.

## Discussion

The aim of the study was to screen the OWH region (Fig. 1) as a TBE high-risk endemic region systematically concerning TBEV prevalence in its main vector *I. ricinus*. The challenge was to overcome the analysis of an area of more than 3530 km^2^ whilst sampling field-collected ticks. Therefore, we have employed a crowdsourcing approach by recruiting and training the interested public to collect ticks in the field using the flagging method [30, 46]. Thus, we were able to successfully implement the project with our limited resources and could reach a grade of sampling coverage up to 78% of estimated grid squares of the total OWH. Crowdsourcing to collect ticks for science has been applied in some other projects in the past to acquire citizen science data [47–49]. To our knowledge, we performed successfully a citizen-based approach for the first time to collect ticks systematically in a complete defined TBE risk region.

To our surprise, the overall TBEV prevalence in *I. ricinus* was unexpectedly low. The prevalence was 0.04% and the minimum infection rate (MIR) was 0.4% for the whole OWH (Table 1). Accordingly, the prevalence depending on a given district was 2.5–128-fold lower than reported in the past from comparable risk areas in Baden-Württemberg and Bavaria [50, 51] and from other regions in Europe [52–55]. This might be due to our approach of systematical collection of a complete risk region covering more than 3500 km^2^. Thus, we had to reduce the sample size per collected area as our research funds were limited. Sample sizes of a single focus from recent publications reporting TBEV prevalence were often larger (approx. 500–1000 ticks/area). However, sampling and calculation in these publications frequently came from natural foci where human TBE cases had been reported before and thus may have caused a statistical bias. However, one recent study from Germany where ticks were collected from the upper Rhine valley (sample size: 4064 ticks) and another crowdsourcing project from Finland from a nationwide tick collection (sample size: 20,000 ticks) calculated similar TBEV MIR rates in *I. ricinus* of 0.1% and 0.2%, respectively [36, 48]. Since it has been suggested that the spatial distribution of TBEV is constricted to certain areas, forming natural foci that are restricted to very limited areas in size (*c.*500 m^2^), our approach might have more likely detected the prevalence of those foci than the frequency of TBEV within a given focus [56]. Thus, the overall prevalence of TBEV within one single focus might be manifold higher.

Regarding the district level within the OWH risk region, the MIR differed significantly from 0% in the NOK district up to 1.2% in the Bergstraße district (Table 1). The reasons behind this are hard to interpret, first due to the overall low prevalence and secondly due to the fact that the foci from different districts had not been characterized in terms of biotic and non-biotic patterns. However, the infection rates reported by the RKI from the five districts over the past ten years strongly correlate (*r* = 0.86) with the TBEV MIR we have found in the respective districts. Thus, fewest TBE cases have been reported in the NOK district (*n* = 4), whereas the highest rate arose in the Bergstraße district (*n* = 39). These coherencies support our hypothesis that our prevalence data reflect more the frequency of TBEV foci within the OWH region rather than real prevalence of TBEV in *I. ricinus* within a single focus.

**Fig. 5:**
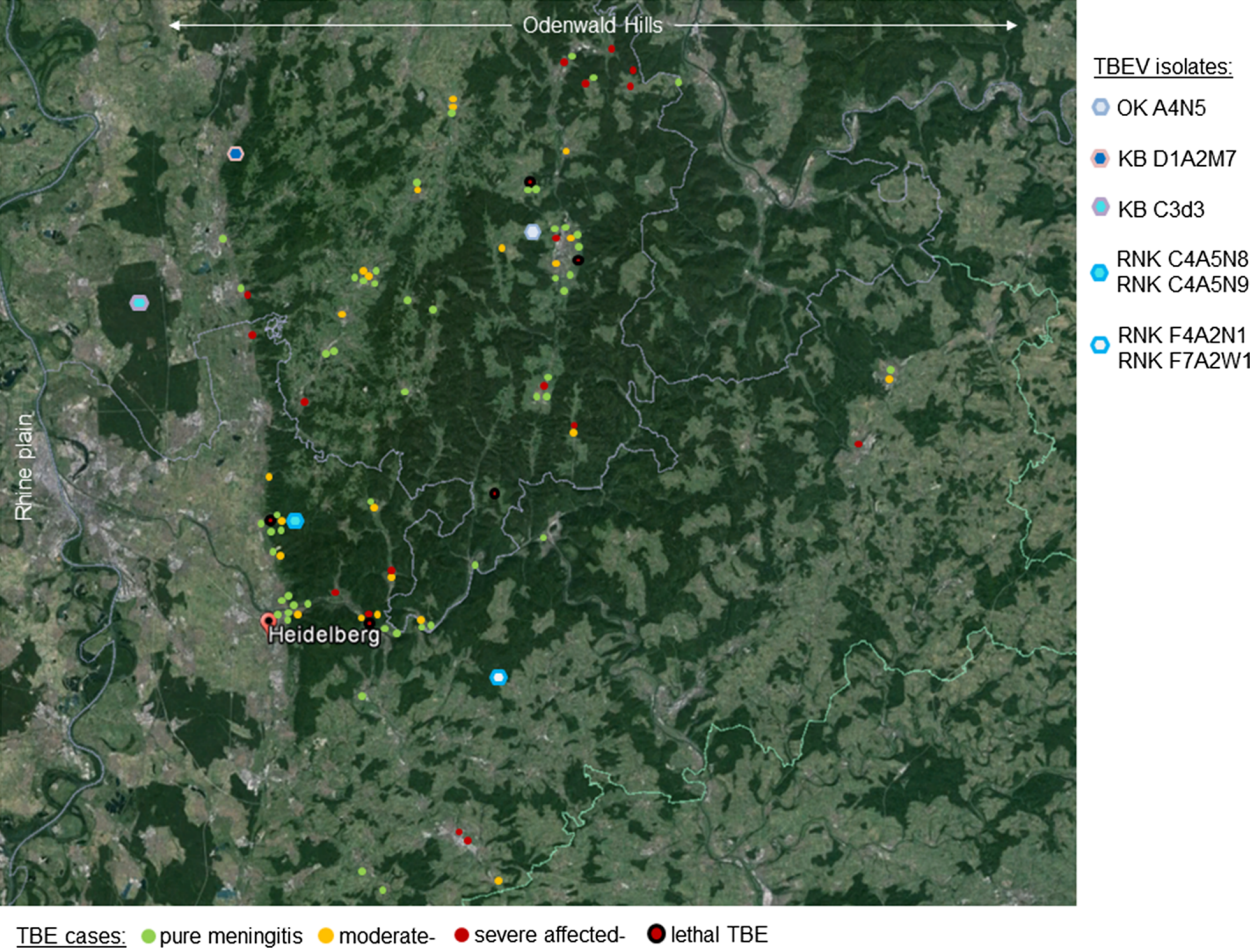
Spatial association of TBEV isolates and reported TBE cases within the OWH. The picture shows the same google earth map section as in Fig. 1. Colored circles show the location of TBE cases reported from 2004 through 2013 and the colored hexagons show the location of the OWH TBEV isolates (hexagons). Note, that isolate OK A4N5 (grey hexagon) and isolates RNK C4A5N8/9 (blue hexagon) are spatially located close to a cluster of frequent and severe to fatal TBE cases, whereas in the proximity of the isolate KB D1A2M7 (dark blue hexagon) no cases have been reported, although the area of isolation is located very close (< 1 km) to a densely populated region at the eastern border of the Rhine plain

One TBEV isolate arose from a single adult *D. reticulatus* tick (OW_KBC3DR3). The tick was collected in an alluvial forest in the middle of the Rhine plain (Fig. 5). The role of *Dermacentor* ticks as transmitting vectors for maintaining the natural cycle in reservoir animals and in TBEV foci has not been definitely clarified [57, 58]. However, quite recently it has been shown that TBEV could be stably isolated from *D. reticulatus* in the absence of *I. ricinus* within a period of three years, indicating that *D. reticulatus* is at least equally important in the maintenance of TBEV in a natural focus [23]. However, whether *D. reticulatus* has the potential to transmit viruses to humans and can cause infections is still unclear.

Phylogenetic analysis of the complete pE gene confirmed that the OWH TBEV isolates belong to the European subtype. The genetic distance within the OWH isolates showed a considerable heterogeneity referred to an area of merely 3500 km^2^ and comparative genetic distance calculated for a selection of European wide strains confirmed this (see Levenshtein plots in Additional file 3: Figure S1 and Additional file 4: Figure S2).

The genetic variance of the OWH isolates may be influenced by bird migration. Since the OWH is bound to the Rhine plain, two different routes of migration may contribute to genetic contamination: (i) birds migrating from south to north over long distances that roost in many places along the Rhine valley; and (ii) an east-western route of intermittently migrating birds in autumn from eastern Europe westwards. This hypothesis is supported by the findings of the MCC and MJ network analysis, showing that isolates form the eastern part of the OWH (Fig. 3, green circle) were more related to isolates from central Bohemia (Czech Republic), whereas OWH isolates collected closer to the Rhine plain (Fig. 3, yellow circle) were more related to Austrian, Swiss and Slovakian strains and one Italian strain. A correlation of TBEV genetics and bird migration has previously been suggested and has finally been proven for the first time by Weidmann et al. in 2013 [25]. Thus, our findings for the OWH region are in principle consistent with recent findings [20, 25, 52]. For the sake of completeness, a less-geographically exposed and isolated cross-section, such as the OWH, may additionally promote genetic spread of TBEV due to mobility of terrestrial reservoir animals. Indeed, various reports in the literature have suggested that this pattern of anthropogenic spread is responsible for both long-distance genetic import (e.g. *via* railway, along roads or river systems) and import into geographically exposed or isolated valleys [25, 52, 59].

The overall genetic variability of the pE gene was low (4.2%) and comparable to recent findings [52]. Comparing the rates of non-synonymous and synonymous substitutions revealed numerous sites in the pE gene under purifying selection; these findings are also in agreement with recent studies [25, 28, 52]. The pE is composed of two regions: the so-called ectodomain consisting of domains I–III and the stem-anchor region. We have sequenced the full-length pE gene from our OWH isolates. In contrast to findings from Swiss isolates, the non-synonymous amino acid substitutions in our OWH isolates mapped exclusively to the pE ectodomain (Fig. 4). Looking more closer to the single substitutions, one amino acid exchange (R346A) may have relevance to the binding affinity to the host receptor (heparan sulphate, HS) since the exchange lies on domain III as binding domain and implies strong changes in van-der-Waals radius as well as in hydrophobicity, polarity and acid dissociation constant of residue (Additional file 5: Table S2). However, more distant amino acid residues 308 and 311 on the upper lateral surface of domain III of TBE complex viruses have been identified as determinants of neurovirulence and neuro-invasiveness [29, 41, 60]. In addition, the R346A substitution was detected in all seven OWH isolates, so that the functional meaning remains uncertain. Nevertheless, the putative meaning of some of the other mutations can carefully be extrapolated from other flaviviruses. Three out of six of our identified amino acid exchanges occurred within a fragment reaching from position 305 to 346. In this part of domain III, mutations have been reported that affect neuro-invasiveness of Japanese encephalitis virus and yellow fever virus [29].

We invested a considerable effort into addressing the quality and reproducibility of our results. First of all, we applied a high-fidelity *Pyrococcus* Taq polymerase (PTP) in our PCR sequencing approach. Based on the PTP specific error frequency, the likelihood for a systematically induced single nucleotide exchange by chance is very low (8.23 × 10^−4^ for our amplicon). Furthermore, we cross-validated our TBEV-positive tested pools, by re-analyzing our isolates, and also our negative tick pools, which were validated at a second laboratory in a blinded fashion. Therefore, our results should reflect the real situation in terms of sequence accuracy in the OWH region.

We recently published a TBE cohort from the OWH [10]. Examining the geographical distribution of case severity and fatality, we found a spatial association of the case frequency as well as of case fatality with three of seven isolates (OKA4N5 and RNKC4A5N8/9, Fig. 5). In contrast, another isolate (KB D1A2M2) was found with a distance of less than 1 km to the eastern Rhine plain and directly adjacent to an urban region with several small cities of more than 80,000 inhabitants. However, so far, no TBE infections have been reported from this region.

Looking once again on the pE gene level, the isolates with a spatial association to frequent and severe to fatal cases showed each one different amino acid substitution that was not detected within the other isolates nor in reference strains such as Neudörfl or Hypr which may be indicative of more virulent TBEV strains. One substitution (A317S) implies a change in polarity and is situated closer to the putative binding site for the host HS. Another substitution (L28M) is located in domain II and implies biochemically a presumptive non-relevant mutation (for biochemical properties of AS-substitutions, see also Additional file 5: Table S2). To our knowledge, mutations within domain II have not been described so far. Nevertheless, these isolates have been identified close to areas where patients have contracted more severe and fatal infection. Furthermore, the general TBE fatality rate in the OWH region (3.6%) is approximately twice as high as described for other regions in Europe, indicating that more virulent strains are circulating in these areas [13, 14, 61]. Whether our identified mutations can effectively contribute to neurovirulence and neuro-invasiveness remains unclear and should be further analyzed *in vitro* and by means of a mouse model.

## Conclusions

TBEV prevalence within the OWH risk regions was found to be comparatively low which is probably due to our methodological approach and may more likely reflect prevalence of TBEV foci. As for other European regions, TBEV genetic data showed a discontinuous phylogeny indicating among others an association with bird migration. Mutations within the pE gene were associated with more frequent, severe and fatal TBE infections in the OWH high-risk region.

## Supplementary information

**Additional file 1: Table S1.** Flagged ticks and distribution of tick stages and tick pools of the OWH region.

**Additional file 2: Alignment S1.** Multiple sequence alignment of pE sequences derived from OWH isolates.

**Additional file 3: Figure S1.** Levenshtein plot of the OWH TBEV isolates.

**Additional file 4: Figure S2.** OWH TBEV isolates within the context of European- and outgroup strains (Levenshtein plot).

**Additional file 5: Table S2.** Biochemical properties of pE mutations detected in OWH TBEV isolates.

## Data Availability

Data supporting the conclusions of this article are included within the article and its additional files. TBEV pE sequencing data were deposited in the GenBank database under the accession numbers shown in Additional file 2: Alignment S1. The remaining datasets used and/or analyzed in the present study are available from the corresponding author upon reasonable request.

## Abbreviations

KB: Bergstraße district
MIR: minimum infection rate
MJ: median-joining
NCBI: National Center for Biotechnology Information
NOK: Neckar-Odenwald district
OW: Odenwald district
OWH: Odenwald hills
pE: envelope protein
RNK: Rhein-Neckar district
TBE: tick-borne encephalitis
TBEV: tick-borne encephalitis virus
