# Supplementary material for: Tick-borne encephalitis virus (TBEV) prevalence in field-collected ticks (Ixodes ricinus) and phylogenetic, structural and virulence analysis in a TBE high-risk endemic area in southwestern Germany
Source: Parasit Vectors. 2020 Jun 11;13:303. doi: 10.1186/s13071-020-04146-7 (PMC7291635; doi:10.1186/s13071-020-04146-7)
Supplement: Supplementary file 1 — Additional file 1: Table S1. Flagged ticks and distribution of tick stages and tick pools of the OWH region. [file 13071_2020_4146_MOESM1_ESM.pdf]

Additional file 1: Table S1. Flagged ticks and distribution of tick stages and tick pools of the Odenwald Hill risk region.

#District	$Nymphae [N]		$Female [N]		$Male [N]		$copulating [N]	
	Ticks	Pools	Ticks	Pools	Ticks	Pools	Ticks	Pools
**OK**	3052	308	285	67	370	84	18	-
**NOK**	4650	470	401	104	467	116	6	-
**RNK**	5965	601	459	116	518	130	20	-
**KB**	1054	108	254	59	290	65	0	-
**total**	**14721**	**1487**	**1399**	**346**	**1645**	**395**	**44**	**-**

#, OWH districts: OK, Odenwaldkreis; NOK, Neckar-Odenwaldkreis; RNK, Rhein-Neckarkreis; KB, Kreis Bergstraße; $, *Ixodes ricinus*, *Dermacentor reticularis* not included.
