# Supplementary material for: Tick-borne encephalitis virus (TBEV) prevalence in field-collected ticks (Ixodes ricinus) and phylogenetic, structural and virulence analysis in a TBE high-risk endemic area in southwestern Germany
Source: Parasit Vectors. 2020 Jun 11;13:303. doi: 10.1186/s13071-020-04146-7 (PMC7291635; doi:10.1186/s13071-020-04146-7)
Supplement: Supplementary file 2 — Additional file 2: Alignment S1. Multiple sequence alignment of pE sequences derived from OWH isolates. [file 13071_2020_4146_MOESM2_ESM.pdf]

Additional file 2: Alignment S1. Multiple sequence alignment of pE sequences from Odenwald Hill isolates.

```
Ref._TBEV_Neudoerfl_pE          -----------------------TCGCGTTGCACACACTTGGAAAACAGGG
Ref._TBEV_Hypr_pE               -----------------------TCGCGTTGCACACATTTGGAAAACAGGG
TBEV_RNKC4A5N9                  GTGTTTAGCACCGGTCTACGCTTCGCGTTGCACACACTTGGAAAACAGGG
TBEV_RNK_C4A5N8                 -----------------------TCGCGTTGCACACACTTGGAAAACAGGG
TBEV_KB_C3d3                    -----------------------TCGCGTTGCACACACTTGGAAAACAGGG
TBEV_RNK_F7A2W1                 -----------------------TCGCGTTGCACACACTTGGAAAACAGGG
TBEV_RNK_F7A2N1                 GTGTCTAGCACCGGTTTACGCTTCGCGTTGCACACACTTGGAAAACAGGG
TBEV_OK_A4N5                    -----------------------TCGCGTTGCACACACTTGGAAAACAGGG
TBEV_KB_D1A2M7                  -----------------------TCACGTTGCACACACTTGGAAAACAGGG
                                                       ** *************************

Ref._TBEV_Neudoerfl_pE          ACTTTGTGACTGGTACTCAGGGGACTACGAGGGTCACCTTGGTGCTGGAA
Ref._TBEV_Hypr_pE               ACTTTGTGACTGGTACTCAGGGGACTACGAGGGTCACCTTGGTGCTGGAA
TBEV_RNKC4A5N9                  ACTTTGTGACTGGTACTCAGGGGACTACGAGGGTCACCTTGGTGCTGGAA
TBEV_RNK_C4A5N8                 ACTTTGTGACTGGTACTCAGGGGACTACGAGGGTCACCTTGGTGCTGGAA
TBEV_KB_C3d3                    ACTTTGTGACTGGTACTCAGGGGACCACGAGAGTCACCTTGGTGCTGGAA
TBEV_RNK_F7A2W1                 ACTTTGTGACTGGTACTCAGGGGACCACGAGAGTCACCTTGGTGCTGGAA
TBEV_RNK_F7A2N1                 ACTTTGTGACTGGTACTCAGGGGACCACGAGAGTCACCTTGGTGCTGGAA
TBEV_OK_A4N5                    ACTTTGTGACTGGTACTCAGGGGACCACGAGGGTCACCTTGGTGCTGGAA
TBEV_KB_D1A2M7                  ACTTTGTGACTGGCACTCAGGGGACCACGAGGGTCACCTTGGTGTTGGAA
                                ************* *********** ***** ************ *****

Ref._TBEV_Neudoerfl_pE          CTGGGTGGATGTGTTACTATAACAGCTGAGGGGAAGCCTTCAATGGATGT
Ref._TBEV_Hypr_pE               CTGGGTGGATGTGTTACCATAACAGCTGAGGGGAAGCCTTCAATGGATGT
TBEV_RNKC4A5N9                  CTGGGTGGATGTGTTACCATAACAGCTGAGGGGAAGCCTTCAATGGATGT
TBEV_RNK_C4A5N8                 CTGGGTGGATGTGTTACCATAACAGCTGAGGGGAAGCCTTCAATGGATGT
TBEV_KB_C3d3                    CTGGGTGGATGTGTTACCATAACAGCTGAGGGGAAGCCTTCAATGGATGT
TBEV_RNK_F7A2W1                 CTGGGTGGATGTGTTACCATAACAGCTGAGGGGAAGCCTTCAATGGATGT
TBEV_RNK_F7A2N1                 CTGGGTGGATGTGTTACCATAACAGCTGAGGGGAAGCCTTCAATGGATGT
TBEV_OK_A4N5                    ATGGGTGGATGTGTTACCATAACAGCTGAGGGGAAGCCTTCAATGGATGT
TBEV_KB_D1A2M7                  CTGGGTGGATGTGTTACCATAACAGCTGAGGGGAAGCCTTCAATGGATGT
                                 *************************************************

Ref._TBEV_Neudoerfl_pE          GTGGCTTGACGCCATTTACCAGGAGAACCCTGCTAAGACACGTGAGTACT
Ref._TBEV_Hypr_pE               GTGGCTTGACGCCATTTACCAGGAGAACCCTGCTAAGACACGTGAGTACT
TBEV_RNKC4A5N9                  GTGGCTTGACGCCATTTACCAGGAGAGCCCTGCTAAGACACGTGAGTACT
TBEV_RNK_C4A5N8                 GTGGCTTGACGCCATTTACCAGGAGAGCCCTGCTAAGACACGTGAGTACT
TBEV_KB_C3d3                    GTGGCTTGACGCCATTTACCAGGAGAGCCCTGCCAAGACACGTGAGTACT
TBEV_RNK_F7A2W1                 GTGGCTTGACGCCATTTACCAGGAGAGCCCTGCCAAGACACGTGAGTACT
TBEV_RNK_F7A2N1                 GTGGCTTGACGCCATTTACCAGGAGAGCCCTGCCAAGACACGTGAGTACT
TBEV_OK_A4N5                    GTGGCTTGATGCCATTTACCAGGAGAGCCCTGCTAAGACTCGTGAGTACT
TBEV_KB_D1A2M7                  GTGGCTTGATGCCATTTACCAGGAGAGCCCTGCTAAGACTCGTGAGTACT
                                ********* **************** ****** ***** **********

Ref._TBEV_Neudoerfl_pE          GTTTACACGCCAAGTTGTCGGACACTAAGGTTGCAGCCAGATGCCCAACA
Ref._TBEV_Hypr_pE               GTTTACACGCCAAGTTGTCGGACACTAAGGTTGCAGCCAGATGCCCAACA
TBEV_RNKC4A5N9                  GTTTACACGCCAAGTTGTCGGACACTAAGGTTGCAGCCAGATGCCCAACG
TBEV_RNK_C4A5N8                 GTTTACACGCCAAGTTGTCGGACACTAAGGTTGCAGCCAGATGCCCAACG
TBEV_KB_C3d3                    GCTTGCACGCCAAGTTGTCGGACACTAAGGTTGCAGCCAGATGCCCAACG
TBEV_RNK_F7A2W1                 GCTTGCATGCCAAGTTGTCGGACACTAAGGTTGCAGCCAGATGCCCAACG
TBEV_RNK_F7A2N1                 GCTTGCACGCCAAGTTGTCGGACACTAAGGTTGCAGCCAGATGCCCAACG
TBEV_OK_A4N5                    GTTTACACGCCAAGTTGTCGGACACTAAGGTTGCAGCCAGATGCCCAACA
TBEV_KB_D1A2M7                  GTTTACACGCCAAGTTGTCGGACACTAAGGTTGCATCCAGATGCCCAACA
                                * ** ** *************************** ************* 

Ref._TBEV_Neudoerfl_pE          ATGGGACCAGCCACTTTGGCTGAAGAACACCAGGGTGGCACAGTGTGTAA
Ref._TBEV_Hypr_pE               ATGGGACCAGCCACTTTGGCTGAAGAACACCAGGGTGGCACAGTGTGCAA
TBEV_RNKC4A5N9                  ATGGGACCAGCCACTTTGGCAGAAGAACACCAGGGTGGCACAGTGTGTAA
TBEV_RNK_C4A5N8                 ATGGGACCAGCCACTTTGGCAGAAGAACACCAGGGTGGCACAGTGTGTAA
TBEV_KB_C3d3                    ATGGGACCAGCTACTTTGGCTGAAGAACACCAGGGTGGCACAGTGTGTAA
TBEV_RNK_F7A2W1                 ATGGGACCAGCTACTTTGGCTGAAGAACACCAGGGTGGCACAGTGTGTAA
TBEV_RNK_F7A2N1                 ATGGGACCAGCTACTTTGGCTGAAGAACACCAGGGTGGCACAGTGTGTAA
TBEV_OK_A4N5                    ATGGGACCAGCCACTTTGGCTGAAGAACACCAGGGTGGCACAGTGTGTAA
TBEV_KB_D1A2M7                  ATGGGACCAGCCACTTTGGCTGAAGAACACCAGGGTGGCACAGTGTGTAA
                                *********** ******** *****************************
```

```
Ref._TBEV_Neudoerfl_pE      GAGAGATCAGAGTGATCGAGGCTGGGGCAACCACTGTGGACTGTTTGGAA
Ref._TBEV_Hypr_pE           GAGAGATCAGAGTGATCGAGGCTGGGGCAACCACTGTGGACTTTTTGGAA
TBEV_RNKC4A5N9              GAGAGATCAGAGTGATCGAGGCTGGGGCAACCACTGTGGACTGTTTGGAA
TBEV_RNK_C4A5N8             GAGAGATCAGAGTGATCGAGGCTGGGGCAACCACTGTGGACTGTTTGGAA
TBEV_KB_C3d3                GAGAGATCAGAGTGATCGAGGCTGGGGCAACCACTGTGGACTGTTTGGAA
TBEV_RNK_F7A2W1             GAGAGATCAGAGTGATCGAGGCTGGGGCAACCACTGTGGACTGTTTGGAA
TBEV_RNK_F7A2N1             GAGAGATCAGAGTGATCGAGGCTGGGGCAACCACTGTGGACTGTTTGGAA
TBEV_OK_A4N5                GAGAGATCAGAGTGATCGAGGCTGGGGCAACCACTGTGGACTGTTTGGAA
TBEV_KB_D1A2M7              GAGAGATCAGAGTGATCGAGGCTGGGGCAACCACTGTGGACTGTTTGGAA
                            **************************************************

Ref._TBEV_Neudoerfl_pE      AGGGTAGCATTGTGGCCTGTGTCAAGGCGGCTTGTGAGGCAAAAAAGAAA
Ref._TBEV_Hypr_pE           AGGGTAGCATTGTGGCCTGTGTCAAGGCGGCTTGTGAGGCAAAAAAGAAA
TBEV_RNKC4A5N9              AGGGTAGCATTGTGGCCTGTGTCAAGGCGGCTTGTGAGGCAAAAAAGAAA
TBEV_RNK_C4A5N8             AGGGTAGCATTGTGGCCTGTGTCAAGGCGGCTTGTGAGGCAAAAAAGAAA
TBEV_KB_C3d3                AGGGTAGCATTGTGGCCTGTGTCAAGGCGGCTTGTGAGGCAAAAAAGAAA
TBEV_RNK_F7A2W1             AGGGTAGCATTGTGGCCTGTGTCAAGGCGGCTTGTGAGGCAAAAAAGAAA
TBEV_RNK_F7A2N1             AGGGTAGCATTGTGGCCTGTGTCAAGGCGGCTTGTGAGGCAAAAAAGAAA
TBEV_OK_A4N5                AGGGTAGCATTGTGGCCTGTGTCAAGGCAGCTTGTGAGGCAAAAAAGAAA
TBEV_KB_D1A2M7              AGGGTAGCATTGTGGCCTGTGTCAAGGCGGCTTGTGAGGCAAAAAAGAAA
                            **************************** *********************

Ref._TBEV_Neudoerfl_pE      GCCACAGGACATGTGTACGACGCCAACAAAATAGTGTACACGGTCAAAGT
Ref._TBEV_Hypr_pE           GCCACAGGACATGTGTACGACGCCAACAAAATAGTGTACACGGTCAAAGT
TBEV_RNKC4A5N9              GCCACAGGACATGTGTACGACGCCAACAAAATAGTGTACACGGTTAAAGT
TBEV_RNK_C4A5N8             GCCACAGGACATGTGTACGACGCCAACAAAATAGTGTACACGGTTAAAGT
TBEV_KB_C3d3                GCCACAGGACATGTGTACGACGCCAACAAAATAGTGTACACGGTCAAAGT
TBEV_RNK_F7A2W1             GCCACAGGACATGTGTACGACGCCAACAAAATAGTGTACACGGTCAAAGT
TBEV_RNK_F7A2N1             GCCACAGGACATGTGTACGACGCCAACAAAATAGTGTACACGGTCAAAGT
TBEV_OK_A4N5                GCCACAGGACATGTGTACGACGCCAACAAAATAGTGTATACGGTCAAAGT
TBEV_KB_D1A2M7              GCCACAGGACATGTGTACGACGCCAACAAAATAGTGTATACGGTCAAAGT
                            ************************************** ***** *****

Ref._TBEV_Neudoerfl_pE      CGAACCACACACGGGAGACTATGTTGCCGCAAACGAGACACATAGTGGGA
Ref._TBEV_Hypr_pE           CGAACCACACACGGGAGACTATGTTGCCGCAAACGAGACACATAGTGGGA
TBEV_RNKC4A5N9              CGAACCACACACGGGAGACTATGTTGCCGCAAACGAGACACATAGTGGGA
TBEV_RNK_C4A5N8             CGAACCACACACGGGAGACTATGTTGCCGCAAACGAGACACATAGTGGGA
TBEV_KB_C3d3                CGAACCACACACGGGAGACTATGTTGCCGCAAACGAGACACATAGTGGGA
TBEV_RNK_F7A2W1             CGAACCACACACGGGAGACTATGTTGCCGCAAACGAGACACATAGTGGGA
TBEV_RNK_F7A2N1             CGAACCACACACGGGAGACTATGTTGCCGCAAACGAGACACATAGTGGGA
TBEV_OK_A4N5                CGAGCCACACACGGGAGACTATGTTGCCGCAAACGAGACACATAGTGGGA
TBEV_KB_D1A2M7              CGAGCCACACACGGGAGACTATGTTGCCGCAAACGAGACACATAGTGGGA
                            *** **********************************************

Ref._TBEV_Neudoerfl_pE      GGAAGACGGCATCCTTCACAATTTCTTCAGAGAAAACCATTTTGACTATG
Ref._TBEV_Hypr_pE           GGAAGACGGCATCCTTCACAGTTTCTTCAGAGAAAACCATTCTGACTATG
TBEV_RNKC4A5N9              GGAAGACGGCTTCCTTCACAGTTTCTTCAGAGAAAACCATTCTGACCATG
TBEV_RNK_C4A5N8             GGAAGACGGCTTCCTTCACAGTTTCTTCAGAGAAAACCATTCTGACCATG
TBEV_KB_C3d3                GGAAGACGGCATCCTTCACAGTCTCTTCAGAGAAAACCATTCTGACTATG
TBEV_RNK_F7A2W1             GGAAGACGGCATCCTTCACAGTCTCTTCAGAGAAAACCATTCTGACTATG
TBEV_RNK_F7A2N1             GGAAGACGGCATCCTTCACAGTCTCTTCAGAGAAAACCATTCTGACTATG
TBEV_OK_A4N5                GGAAGACGGCATCCTTCACAGTTTCTTCAGAGAAAACCATTCTGACTATG
TBEV_KB_D1A2M7              GGAAGACGGCATCCTTCACAGTTTCTTCAGAGAAAACCATTCTGACTATG
                            ********** *********** ****************** **** ***

Ref._TBEV_Neudoerfl_pE      GGTGAGTATGGAGATGTGTCTTTGTTGTGCAGGGTCGCTAGTGGCGTT
Ref._TBEV_Hypr_pE           GGTGAGTATGGAGATGTGTCTCTGTTGTGTAGGGTCGCTAGTGGCGTT
TBEV_RNKC4A5N9              GGTGAGTATGGAGATGTGTCTTTGTTGTGCAGGGTCGCTAGTGGCGTG
TBEV_RNK_C4A5N8             GGTGAGTATGGAGATGTGTCTTTGTTGTGCAGGGTCGCTAGTGGCGTG
TBEV_KB_C3d3                GGTGAGTATGGAGATGTGTCCTTGTTGTGCAGGGTTGCTAGTGGCGTT
TBEV_RNK_F7A2W1             GGTGAGTATGGAGATGTGTCCTTGTTGTGCAGGGTTGCTAGTGGCGTT
TBEV_RNK_F7A2N1             GGTGAGTATGGAGATGTGTCCTTGTTGTGCAGGGTTGCTAGTGGCGTT
TBEV_OK_A4N5                GGTGAGTATGGAGATGTGTCTTTGTTGTGCAGGGTCGCCAGCGGCGTT
TBEV_KB_D1A2M7              GGTGAGTATGGAGATGTGTCTTTGTTGTGCAGGGTCGCCAGCGGCGTT
                            ******************** ************** ** ** *****
```

```
Ref._TBEV_Neudoerfl_pE      GACTTGGCCCAGACCGTCATCCTTGAGCTTGACAAGACAGTGGAACACCT
Ref._TBEV_Hypr_pE           GACTTGGCCCAGACCGTCATCCTTGAGCTTGACAAGACAGTGGAACACCT
TBEV_RNKC4A5N9              GACTTGGCTCAGACCGTTATCCTTGAGCTTGACAAGACAGTGGAACACCT
TBEV_RNK_C4A5N8             GACTTGGCTCAGACCGTTATCCTTGAGCTTGACAAGACAGTGGAACACCT
TBEV_KB_C3d3                GACTTGGCCCAAACTGTCATTCTTGAGCTTGACAAGACAGTGGAACACCT
TBEV_RNK_F7A2W1             GACTTGGCCCAAACTGTCATTCTTGAGCTTGACAAGACAGTGGAACACCT
TBEV_RNK_F7A2N1             GACTTGGCCCAAACTGTCATTCTTGAGCTTGACAAGACAGTGGAACACCT
TBEV_OK_A4N5                GACTTGGCCCAGACCGTCATCCTTGAGCTTGACAAGACAGTGGAACACCT
TBEV_KB_D1A2M7              GACTTGGCCCAGACCGTCATCCTTGAGCTTGACAAGACAGTGGAACACCT
                            ******** ** ** ** ** *****************************

Ref._TBEV_Neudoerfl_pE      TCCAACGGCTTGGCAGGTCCATAGGGACTGGTTCAATGATCTGGCTCTG
Ref._TBEV_Hypr_pE           TCCAACGGCTTGGCAGGTCCACAGGGACTGGTTTAATGATCTGGCTCTG
TBEV_RNKC4A5N9              TCCAACGGCTTGGCAGGTCCACAGGGACTGGTTCAATGATCTGGCTCTG
TBEV_RNK_C4A5N8             TCCAACGGCTTGGCAGGTCCACAGGGACTGGTTCAATGATCTGGCTCTG
TBEV_KB_C3d3                TCCAACGGCTTGGCAGGTCCACAGGGACTGGTTCAATGATCTGGCTCTG
TBEV_RNK_F7A2W1             TCCAACGGCTTGGCAGGTCCACAGGGACTGGTTCAATGATCTGGCTCTG
TBEV_RNK_F7A2N1             TCCAACGGCTTGGCAGGTCCACAGGGACTGGTTCAATGATCTGGCTCTG
TBEV_OK_A4N5                TCCAACGGCTTGGCAGGTCCACAGGGACTGGTTCAATGATTTGGCTCTG
TBEV_KB_D1A2M7              TCCAACGGCTTGGCAGGTCCACAGGGACTGGTTCAATGATTTGGCTCTG
                            **************************************** ********

Ref._TBEV_Neudoerfl_pE      CCATGGAAACATGAGGGAGCGCAAAACTGGAACAACGCAGAAAGACTGGT
Ref._TBEV_Hypr_pE           CCATGGAAACATGAGGGAGCGCAAAACTGGAATAACGCAGAAAGATTGGT
TBEV_RNKC4A5N9              CCATGGAAACATGAGGGAGCGCAAAACTGGAACAACGCAGAAAGACTGGT
TBEV_RNK_C4A5N8             CCATGGAAACATGAGGGAGCGCAAAACTGGAACAACGCAGAAAGACTGGT
TBEV_KB_C3d3                CCGTGGAAACATGAGGGAGCGCAAAACTGGAATAACGCAGAAAGACTGGT
TBEV_RNK_F7A2W1             CCGTGGAAACATGAGGGAGCGCAAAACTGGAATAACGCAGAAAGACTGGT
TBEV_RNK_F7A2N1             CCGTGGAAACATGAGGGAGCGCAAAACTGGAATAACGCAGAAAGACTGGT
TBEV_OK_A4N5                CCATGGAAACATGAGGGAGCGCAAAACTGGAACAACGCAGAAAGACTGGT
TBEV_KB_D1A2M7              CCATGGAAACATGAGGGAGCGCAAAACTGGAACAACGCAGAAAGACTGGT
                            ** ***********************************************

Ref._TBEV_Neudoerfl_pE      TGAATTTGGGGCTCCTCACGCTGTCAAGATGGACGTGTACAACCTCGGAG
Ref._TBEV_Hypr_pE           TGAATTTGGGGCTCCTCATGCTGTCAAGATGGATGTGTACAACCTCGGAG
TBEV_RNKC4A5N9              TGAATTTGGGGCTCCGCACGCTGTCAAGATGGACGTGTATAACCTCGGAG
TBEV_RNK_C4A5N8             TGAATTTGGGGCTCCGCACGCTGTCAAGATGGACGTGTATAACCTCGGAG
TBEV_KB_C3d3                TGAATTTGGGGCTCCTCACGCTGTCAAGATGGACGTGTACAACCTCGGAG
TBEV_RNK_F7A2W1             TGAATTTGGGGCTCCTCACGCTGTCAAGATGGACGTGTACAACCTCGGAG
TBEV_RNK_F7A2N1             TGAATTTGGGGCTCCTCACGCTGTCAAGATGGACGTGTACAACCTCGGAG
TBEV_OK_A4N5                TGAATTTGGGGCTCCTCACGCTGTCAAGATGGACGTGTACAACCTCGGAG
TBEV_KB_D1A2M7              TGAATTTGGGGCTCCTCACGCTGTCAAGATGGACGTGTACAACCTCGGAG
                            *************** **********************************

Ref._TBEV_Neudoerfl_pE      ACCAGACTGGAGTGTTACTGAAGGCTCTCGCTGGGGTTCCTGTGGCACAC
Ref._TBEV_Hypr_pE           ACCAGACTGGAGTGTTACTGAAGGCTCTCGCTGGGGTTCCTGTGGCACAC
TBEV_RNKC4A5N9              ACCAGACTGGAGTGTTACTGAAGGCTCTCGCTGGGGTTCCTGTGGCACAC
TBEV_RNK_C4A5N8             ACCAGACTGGAGTGTTACTGAAGGCTCTCGCTGGGGTTCCTGTGGCACAC
TBEV_KB_C3d3                ACCAGACTGGAGTGTTACTGAAGGCTCTCGCTGGGGTTCCTGTGGCACAC
TBEV_RNK_F7A2W1             ACCAGACTGGAGTGTTACTGAAGGCTCTCGCTGGGGTTCCTGTGGCACAC
TBEV_RNK_F7A2N1             ACCAGACTGGAGTGTTACTGAAGGCTCTCGCTGGGGTTCCTGTGGCACAC
TBEV_OK_A4N5                ACCAGACTGGAGTGTTACTGAAGGCTCTCGCTGGGGTTCCTGTGGCACAC
TBEV_KB_D1A2M7              ACCAGACTGGAGTGTTGCTGAAGGCTCTCGCTGGGGTTCCTGTGGCACAC
                            **************** *********************************

Ref._TBEV_Neudoerfl_pE      ATTGAGGGAACCAAGTACCACCTGAAGAGTGGCCACGTGACCTGCGAAGT
Ref._TBEV_Hypr_pE           ATTGAGGGAACCAAGTACCACCTGAAGAGTGGCCATGTGACCTGCGAAGT
TBEV_RNKC4A5N9              ATTGAGGGAACCAAGTACCACCTGAAGAGTGGCCATGTGACATGCGAAGT
TBEV_RNK_C4A5N8             ATTGAGGGAACCAAGTACCACCTGAAGAGTGGCCATGTGACATGCGAAGT
TBEV_KB_C3d3                ATTGAGGGAACCAAGTACCACCTGAAGAGTGGCCATGTGACCTGCGAAGT
TBEV_RNK_F7A2W1             ATTGAGGGAACCAAGTACCACCTGAAGAGTGGCCATGTGACCTGCGAAGT
TBEV_RNK_F7A2N1             ATTGAGGGAACCAAGTACCACCTGAAGAGTGGCCATGTGACCTGCGAAGT
TBEV_OK_A4N5                ATTGAGGGAACTAAGTACCACCTGAAGAGTGGCCATGTGACCTGCGAAGT
TBEV_KB_D1A2M7              ATTGAGGGAACTAAGTACCACCTGAAGAGTGGCCATGTGACCTGCGAAGT
                            *********** ***************************** ********
```

```
Ref._TBEV_Neudoerfl_pE        GGGACTGGAAAAACTGAAGATGAAAGGTCTTACGTACACAATGTGTGACA
Ref._TBEV_Hypr_pE             GGGACTGGAAAAACTGAAGATGAAAGGTCTTACGTACACAATGTGTGACA
TBEV_RNKC4A5N9                GGGACTGGAAAAACTGAAGATGAAAGGTCTTACGTACACAATGTGTGACA
TBEV_RNK_C4A5N8               GGGACTGGAAAAACTGAAGATGAAAGGTCTTACGTACACAATGTGTGACA
TBEV_KB_C3d3                  GGGACTGGAAAAACTGAAGATGAAAGGTCTCACGTACATAATGTGTGACA
TBEV_RNK_F7A2W1               GGGACTGGAAAAACTGAAGATGAAAGGTCTCACGTACATAATGTGTGACA
TBEV_RNK_F7A2N1               GGGACTGGAAAAACTGAAGATGAAAGGTCTCACGTACATAATGTGTGACA
TBEV_OK_A4N5                  GGGACTGGAAAAACTGAAGATGAAAGGTCTCACGTACACAATGTGTGACA
TBEV_KB_D1A2M7                GGGACTGGAAAAACTGAAGATGAAAGGTCTCACGTACACAATGTGTGACA
                              ****************************** ******* ***********

Ref._TBEV_Neudoerfl_pE        AAACAAAGTTCACATGGAAGAGAGCTCCAACAGACAGTGGGCATGATACA
Ref._TBEV_Hypr_pE             AAACAAAGTTCACATGGAAGAGAGCTCCAACAGACAGTGGGCATGATACA
TBEV_RNKC4A5N9                AAACAAAGTTCACATGGAAGAGAGTTCCAACAGACAGTGGACATGATACA
TBEV_RNK_C4A5N8               AAACAAAGTTCACATGGAAGAGAGTTCCAACAGACAGTGGACATGATACA
TBEV_KB_C3d3                  AAACAAAGTTCACATGGAAGAGAGCTCCAACAGATAGTGGGCATGATACA
TBEV_RNK_F7A2W1               AAACAAAGTTCACATGGAAGAGAGCTCCAACAGATAGTGGGCATGATACA
TBEV_RNK_F7A2N1               AAACAAAGTTCACATGGAAGAGAGCTCCAACAGATAGTGGGCATGATACA
TBEV_OK_A4N5                  AAACAAAGTTCACATGGAAGAGAGCTCCAACAGACAGTGGGCATGATACA
TBEV_KB_D1A2M7                AAACAAAGTTCACATGGAAGAGAGCTCCAACAGACAGTGGGCATGATACA
                              ************************ ********* ***** *********

Ref._TBEV_Neudoerfl_pE        GTGGTCATGGAAGTCACATTCTCTGGAACAAAGCCCTGTAGGATCCCAGT
Ref._TBEV_Hypr_pE             GTGGTCATGGAAGTCACATTCTCTGGAACAAAGCCCTGTAGGATCCCAGT
TBEV_RNKC4A5N9                GTGGTCATGGAAGTCACATTCTCTGGAACAAAGCCCTGCAGGATCCCAGT
TBEV_RNK_C4A5N8               GTGGTCATGGAAGTCACATTCTCTGGAACAAAGCCCTGCAGGATCCCAGT
TBEV_KB_C3d3                  GTGGTCATGGAAGTCACATTCTCTGGAACAAAGCCCTGTAGGATCCCAGT
TBEV_RNK_F7A2W1               GTGGTCATGGAAGTCACATTCTCTGGAACAAAGCCCTGTAGGATCCCAGT
TBEV_RNK_F7A2N1               GTGGTCATGGAAGTCACATTCTCTGGAACAAAGCCCTGTAGGATCCCAGT
TBEV_OK_A4N5                  GTGGTCATGGAAGTCACATTCTCTGGAACAAAGCCCTGCAGGATCCCAGT
TBEV_KB_D1A2M7                GTGGTCATGGAAGTCACATTCTCTGGAACAAAGCCCTGCAGGATCCCAGT
                              **************************************************

Ref._TBEV_Neudoerfl_pE        CAGGGCAGTGGCACATGGATCTCCAGATGTGAACGTGGCCATGCTGATAA
Ref._TBEV_Hypr_pE             CAGGGCAGTGGCACATGGATCTCCAGATGTGAACGTGGCCATGCTGATAA
TBEV_RNKC4A5N9                CAGGGCAGTGGCACATGGATCTCCAGATGTGAACGTGGCCATGCTGATAA
TBEV_RNK_C4A5N8               CAGGGCAGTGGCACATGGATCTCCAGATGTGAACGTGGCCATGCTGATAA
TBEV_KB_C3d3                  CAGGGCAGTGGCACATGGATCTCCAGATGTGAATGTGGCCATGCTGATAA
TBEV_RNK_F7A2W1               CAGGGCAGTGGCACATGGATCTCCAGATGTGAATGTGGCCATGCTGATAA
TBEV_RNK_F7A2N1               CAGGGCAGTGGCACATGGATCTCCAGATGTGAATGTGGCCATGCTGATAA
TBEV_OK_A4N5                  CAGGGCAGTGGCACATGGATCTCCAGATGTGAACGTGGCCATGCTGATAA
TBEV_KB_D1A2M7                CAGGGCAGTGGCACATGGATCTCCAGATGTGAACGTGGCCATGCTGATAA
                              **************************************************

Ref._TBEV_Neudoerfl_pE        CGCCAAACCCAACAATTGAAAACAATGGAGGTGGCTTCATAGAGATGCAG
Ref._TBEV_Hypr_pE             CGCCAAACCCAACAATTGAAAACAATGGAGGTGGCTTCATAGAGATGCAG
TBEV_RNKC4A5N9                CGCCAAACCCAACAATTGAAAACAATGGAGGTGGCTTCATAGAGATGCAG
TBEV_RNK_C4A5N8               CGCCAAACCCAACAATTGAAAACAATGGAGGTGGCTTCATAGAGATGCAG
TBEV_KB_C3d3                  CGCCAAACCCAACAATTGAAAACAATGGAGGTGGCTTCATAGAGATGCAG
TBEV_RNK_F7A2W1               CGCCAAACCCAACAATTGAAAACAATGGAGGTGGCTTCATAGAGATGCAG
TBEV_RNK_F7A2N1               CGCCAAACCCAACAATTGAAAACAATGGAGGTGGCTTCATAGAGATGCAG
TBEV_OK_A4N5                  CGCCAAACCCAACAATTGAAAACAATGGAGGTGGCTTCATAGAGATGCAG
TBEV_KB_D1A2M7                CGCCAAACCCAACAATTGAAAACAATGGAGGTGGCTTCATAGAGATGCAG
                              **************************************************

Ref._TBEV_Neudoerfl_pE        CTGCCCCCAGGGGATAACATCATCTATGTTGGGGAACTGAGTCATCAATG
Ref._TBEV_Hypr_pE             CTGCCCCCAGGGGACAACATCATCTATGTTGGGGAACTGAGTCATCAATG
TBEV_RNKC4A5N9                CTGCCCCCAGGGGATAACATCATCTATGTTGGGGAACTGAGTCATCAATG
TBEV_RNK_C4A5N8               CTGCCCCCAGGGGATAACATCATCTATGTTGGGGAACTGAGTCATCAATG
TBEV_KB_C3d3                  CTGCCCCCAGGGGACAACATTATCTATGTTGGGGAACTGAGTCATCAATG
TBEV_RNK_F7A2W1               CTGCCCCCAGGGGACAACATTATCTATGTTGGGGAACTGAGTCATCAATG
TBEV_RNK_F7A2N1               CTGCCCCCAGGGGACAACATTATCTATGTTGGGGAACTGAGTCATCAATG
TBEV_OK_A4N5                  CTGCCCCCAGGTGATAACATCATCTATGTTGGGGAACTGAGTCATCAATG
TBEV_KB_D1A2M7                CTGCCCCCAGGTGATAACATCATCTATGTTGGGGAACTGAGTCATCAATG
                              *********** ******** *****************************

Ref._TBEV_Neudoerfl_pE        GTTCCAAAAAGGGAGCAGCATCGGAAGGGTTTTCCAAAAGACCAAGAAAG
```

```
Ref._TBEV_Hypr_pE            GTTCCAAAAAGGGAGCAGTATCGGAAAGGTTTTCCAAAAGACCAAGAAAG
TBEV_RNKC4A5N9               GTTCCAAAAAGGGAGCAGCATCGGAAGGGTTTTCCAAAAGACCAAGAAAG
TBEV_RNK_C4A5N8              GTTCCAAAAAGGGAGCAGCATCGGAAGGGTTTTCCAAAAGACCAAGAAAG
TBEV_KB_C3d3                 GTTCCAAAAAGGGAGCAGCATCGGAAGGGTTTTTCAAAAGACCAAGAAAG
TBEV_RNK_F7A2W1              GTTCCAAAAAGGGAGCAGCATCGGAAGGGTTTTTCAAAAGACCAAGAAAG
TBEV_RNK_F7A2N1              GTTCCAAAAAGGGAGCAGCATCGGAAGGGTTTTTCAAAAGACCAAGAAAG
TBEV_OK_A4N5                 GTTCCAAAAAGGGAGCAGCATCGGAAGGGTTTTCCAAAAGACCAAGAAGG
TBEV_KB_D1A2M7               GTTCCAAAAAGGGAGTAGCATCGGAAGGGTTTTCCAAAAGACCAAGAAAG
                             *************** **************** *************** *

Ref._TBEV_Neudoerfl_pE       GCATAGAAAGACTGACAGTGATAGGAGAGCACGCCTGGGACTTCGGTTCT
Ref._TBEV_Hypr_pE            GCATAGAAAGACTGACAGTGATAGGGGAGCACGCCTGGGACTTCGGTTCT
TBEV_RNKC4A5N9               GCATAGAAAGACTGACAGTGATAGGAGAGCACGCCTGGGACTTTGGTTCT
TBEV_RNK_C4A5N8              GCATAGAAAGACTGACAGTGATAGGAGAGCACGCCTGGGACTTTGGTTCT
TBEV_KB_C3d3                 GCATAGAAAGACTGACAGTGATAGGAGAGCACGCCTGGGACTTCGGTTCT
TBEV_RNK_F7A2W1              GCATAGAAAGACTGACAGTGATAGGAGAGCACGCCTGGGACTTCGGTTCT
TBEV_RNK_F7A2N1              GCATAGAAAGACTGACAGTGATAGGAGAGCACGCCTGGGACTTCGGTTCT
TBEV_OK_A4N5                 GCATAGAAAGACTGACAGTGATAGGAGAGCACGCCTGGGACTTTGGTTCT
TBEV_KB_D1A2M7               GCATAGAAAGACTGACAGTGATAGGAGAGCACGCCTGGGACTTTGGTTCT
                             ******************************************* ******

Ref._TBEV_Neudoerfl_pE       GCTGGAGGCTTTCTGAGTTCAATTGGGAAGGCGGTACATACGGTCCTTGG
Ref._TBEV_Hypr_pE            GCTGGAGGCTTTCTGAGTTCAATTGGGAAGGCGGTGCACACGGTCCTTGG
TBEV_RNKC4A5N9               GCTGGAGGCTTTCTGAGTTCAATTGGGAAGGCGGTGCACACAGTCCTTGG
TBEV_RNK_C4A5N8              GCTGGAGGCTTTCTGAGTTCAATTGGGAAGGCGGTGCACACAGTCCTTGG
TBEV_KB_C3d3                 GCTGGAGGCTTTCTGAGTTCAATTGGGAAGGCGGTGCACACGGTCCTTGG
TBEV_RNK_F7A2W1              GCTGGAGGCTTTCTGAGTTCAATTGGGAAGGCGGTGCACACGGTCCTTGG
TBEV_RNK_F7A2N1              GCTGGAGGCTTTCTGAGTTCAATTGGGAAGGCGGTGCACACGGTCCTTGG
TBEV_OK_A4N5                 GCTGGAGGCTTTCTGAGTTCAATTGGGAAGGTGGTGCATACGGTCCTTGG
TBEV_KB_D1A2M7               GCTGGAGGCTTTCTGAGTTCAATTGGGAAGGCGGTGCATACGGTCCTTGG
                             ******************************* ****** ** ********

Ref._TBEV_Neudoerfl_pE       TGGCGCTTTCAACAGCATCTTCGGGGGAGTGGGGTTTCTACCAAAACTTT
Ref._TBEV_Hypr_pE            TGGTGCTTTCAACAGCATCTTCGGGGGAGTGGGGTTTCTACCAAAGCTTC
TBEV_RNKC4A5N9               TGGTGCTTTTAACAGCATCTTCGGGGGAGTGGGGTTTCTGCCAAAGCTTT
TBEV_RNK_C4A5N8              TGGTGCTTTTAACAGCATCTTCGGGGGAGTGGGGTTTCTGCCAAAGCTTT
TBEV_KB_C3d3                 TGGTGCTTTCAACAGCATCTTCGGGGGAGTAGGGTTTCTACCAAAGCTTT
TBEV_RNK_F7A2W1              TGGTGCTTTCAACAGCATCTTCGGGGGAGTAGGGTTTCTACCAAAGCTTT
TBEV_RNK_F7A2N1              TGGTGCTTTCAACAGCATCTTCGGGGGAGTAGGGTTTCTACCAAAGCTTT
TBEV_OK_A4N5                 TGGTGCTTTCAACAGCATCTTCGGGGGAGTGGGGTTTCTGCCAAAGCTTC
TBEV_KB_D1A2M7               TGGTGCTTTCAACAGCATCTTTGGGGGAGTGGGGTTTCTGCCAAAGCTTC
                             ********* *********** ******** ******** **********

Ref._TBEV_Neudoerfl_pE       TATTAGGAGTGGCATTGGCTTGGTTGGGCCTGAACATGAGAAACCCTACA
Ref._TBEV_Hypr_pE            TATTAGGAGTGGCATTGGCTTGGTTGGGCCTGAACATGAGAAACCCTACA
TBEV_RNKC4A5N9               TACTAGGAGTGGCATTGGCTTGGTTGGGCCTGAACATGAGAAACCCTACA
TBEV_RNK_C4A5N8              TACTAGGAGTGGCATTGGCTTGGTTGGGCCTGAACATGAGAAACCCTACA
TBEV_KB_C3d3                 TATTAGGAGTGGCATTGGCTTGGTTGGGCCTGAACATGAGAAACCCTACA
TBEV_RNK_F7A2W1              TATTAGGAGTGGCATTGGCTTGGTTGGGCCTGAACATGAGAAACCCTACA
TBEV_RNK_F7A2N1              TATTAGGAGTGGCATTGGCTTGGTTGGGCCTGAACATGAGAAACCCTACA
TBEV_OK_A4N5                 TATTAGGAGTGGCATTGGCCTGGTTGGGGCTGAACATGAGAAACCCTACA
TBEV_KB_D1A2M7               TATTAGGAGTGGCATTGGCCTGGTTGGGGCTGAACATGAGAAACCCTACA
                             ** **************** ******** *********************

Ref._TBEV_Neudoerfl_pE       ATGTCCATGAGCTTTCTCTTGGCTGGAGGTCTGGTCTTGGCCATGACCCT
Ref._TBEV_Hypr_pE            ATGTCCATGAGCTTTCTCTTGGCTGGAGTTCTGGTTTTGGCCATGACCCT
TBEV_RNKC4A5N9               ATGTCCATGAGCTTTCTCTTGGCTGGAGGTCTGGTCTTGGCCATGACCCT
TBEV_RNK_C4A5N8              ATGTCCATGAGCTTTCTCTTGGCTGGAGGTCTGGTCTTGGCCATGACCCT
TBEV_KB_C3d3                 ATGTCCATGAGCTTTCTCTTGGCTGGAGGTCTGGTCTTGGCCATGACCCT
TBEV_RNK_F7A2W1              ATGTCCATGAGCTTTCTCTTGGCTGGAGGTCTGGTCTTGGCCATGACCCT
TBEV_RNK_F7A2N1              ATGTCCATGAGCTTTCTCTTGGCTGGAGGTCTGGTCTTGGCCATGACCCT
TBEV_OK_A4N5                 ATGTCCATGAGCTTTCTCCTGGCTGGAGGTCTGGTCTTGGCCATGACCCT
TBEV_KB_D1A2M7               ATGTCCATGAGCTTTCTCCTGGCTGGAGGTCTGGTCTTGGCCATGACCCT
                             ****************** *******************************
```

```
Ref._TBEV_Neudoerfl_pE       TGGAGTGGGGGCG-------------------------------
Ref._TBEV_Hypr_pE            TGGAGTGGGGGCG-------------------------------
TBEV_RNKC4A5N9               TGGAGTGGGGGCGGATGTTGGCTGCGCTGTGGACACGAACGAAG
TBEV_RNK_C4A5N8              TGGAGTG-------------------------------------
TBEV_KB_C3d3                 TGGAGTG-------------------------------------
TBEV_RNK_F7A2W1              TGGAGTG-------------------------------------
TBEV_RNK_F7A2N1              TGGAGTGGGGGCGGATGTTGGCTGCGCTGTGGACACG-------
TBEV_OK_A4N5                 TGGAGTG-------------------------------------
TBEV_KB_D1A2M7               CGGAGTG-------------------------------------
                              ******
```

CLUSTAL W (1.81) multiple sequence alignment, consensus key:

* - single, fully conserved residue

: - conservation of strong groups

. - conservation of weak groups

  - no consensus

  - single nucleotide exchanges

Ref.: reference strains Neudörfl (GenBank, AC: U27495) and Hypr (GenBank, AC: U39292)(yellow area).

Nomenclature of OWH isolates (grey area): TBEV_district_coordinate of grid square and N, *Ixodes* nympha, M, *Ixodes* male, W, *Ixodes* female, d, *Dermacentor reticulatus*, and number of pool (*Ixodes*) or individual tick (*Dermacentor*).

GenBank AC numbers for the isolates:

TBEV_KB_D1A2M7: MT181019; TBEV_RNK_F7A2W1: MT181020; TBEV_OK_A4N5: MT181021; TBEV_RNK_C4A5N8: MT181022; TBEV_KB_C3d3: MT181023; TBEV_RNKC4A5N9. MT181024; TBEV_RNK_F7A2N1: MT181025.
