# Supplementary figures and images for: Tick-borne encephalitis virus (TBEV) prevalence in field-collected ticks (Ixodes ricinus) and phylogenetic, structural and virulence analysis in a TBE high-risk endemic area in southwestern Germany
Source: Parasit Vectors. 2020 Jun 11;13:303. doi: 10.1186/s13071-020-04146-7 (PMC7291635; doi:10.1186/s13071-020-04146-7)

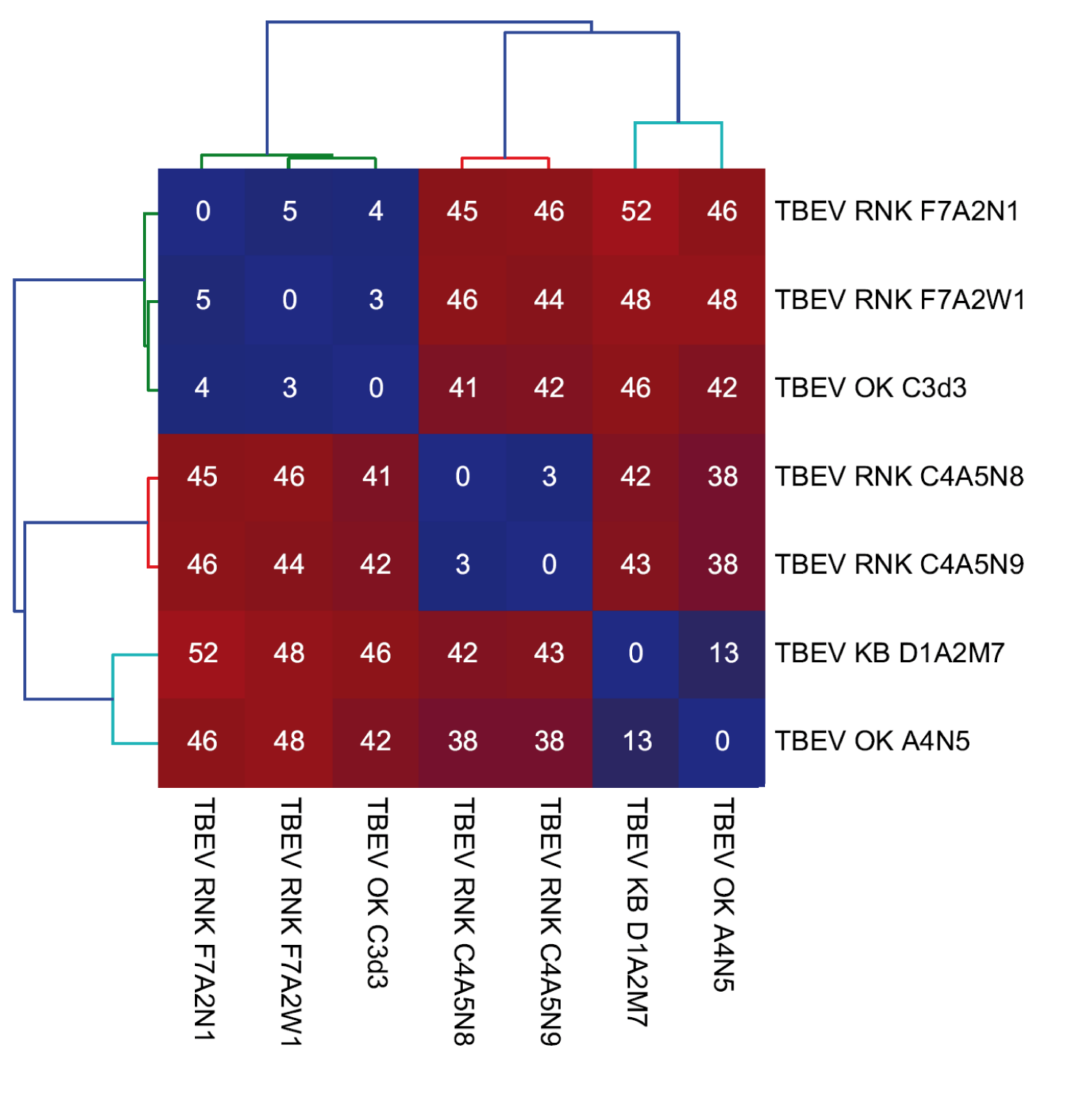

Supplement: Supplementary file 3 — Additional file 3: Figure S1. Levenshtein plot of the OWH TBEV isolates. [file 13071_2020_4146_MOESM3_ESM.tif]

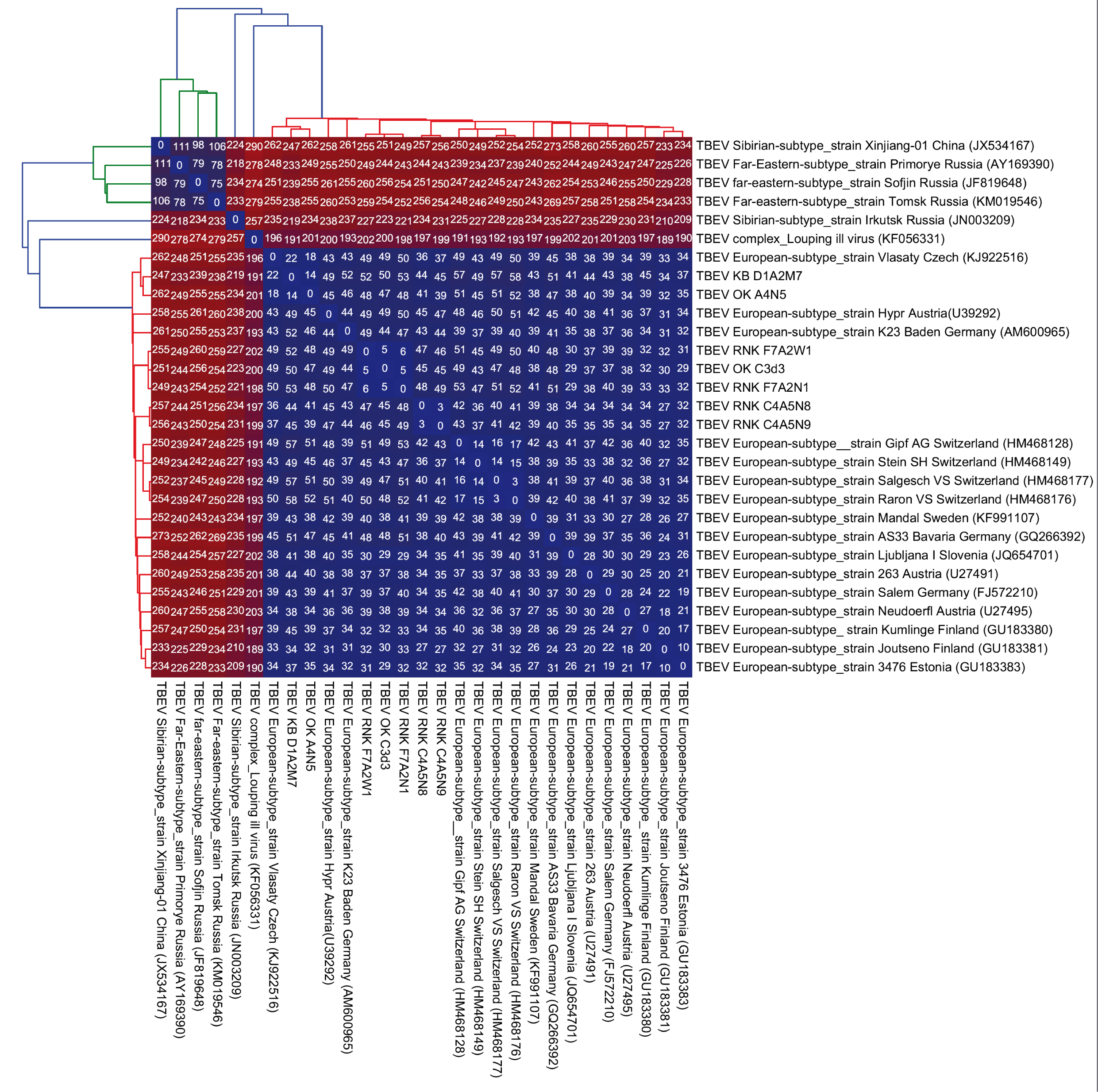

Supplement: Supplementary file 4 — Additional file 4: Figure S2. OWH TBEV isolates within the context of European- and outgroup strains (Levenshtein plot). [file 13071_2020_4146_MOESM4_ESM.tif]
