# Supplementary material for: Tick-borne encephalitis virus (TBEV) prevalence in field-collected ticks (Ixodes ricinus) and phylogenetic, structural and virulence analysis in a TBE high-risk endemic area in southwestern Germany
Source: Parasit Vectors. 2020 Jun 11;13:303. doi: 10.1186/s13071-020-04146-7 (PMC7291635; doi:10.1186/s13071-020-04146-7)
Supplement: Supplementary file 5 — Additional file 5: Table S2. Biochemical properties of pE mutations detected in OWH TBEV isolates. [file 13071_2020_4146_MOESM5_ESM.pdf]

Additional file 5: Table S2. Biochemical properties of pE mutations detected in OWH TBEV isolates.

Mutation	Side-chain	Van-d.-Waals-Volume	Hydrophobicity[$]	Acidity	Acid dissociation constant
**AS28:L→M (domain II)**					
Leucine (L)	CH2CH(CH3)2	124	3,8	neutral	-
Methionine (M)	CH2CH2SCH3	124	1,9	neutral	-
**AS52:N→S (domain I)**					
Asparagine (N)	CH2CONH2	96	-3.5	neutral	-
Serine (S)	CH2OH	73	-0.8	neutral	-
**AS72 :A→S (domain I)**					
Alanine (A)	CH3	67	1.8	neutral	-
Serine (S)	CH2OH	73	-0.8	neutral	-
**AS305:T→I (domain III)**					
Threonine (T)	CH(OH)CH3	93	-0.7	neutral	-
Isoleucine (I)	CH(CH3)CH2CH3	124	4.5	neutral	-
**AS317:A→S (domain III)**					
Alanine (A)	CH3	67	1.8	neutral	-
Serine (S)	CH2OH	73	-0.8	neutral	-
**AS346:R→A (domain III)**					
Arginine (R)	CH2CH2CH2NH-C(NH)NH2	143	-4.5	basic	12.48
Alanine (A)	CH3	67	1.8	neutral	-

$, hydrophobicity: < 0 ‘polar’, > 0 ‘unpolar’ residues; AS, amino acid residue of the pE chain.

W. R. Taylor: The classification of amino acid conservation. In: Journal of Theoretical Biology, 1986, 119: 205–218.
